# Unraveling Hidden Components of the Chloroplast Envelope Proteome: Opportunities and Limits of Better MS Sensitivity*[Fn FN2]

**DOI:** 10.1074/mcp.RA118.000988

**Published:** 2019-04-08

**Authors:** Imen Bouchnak, Sabine Brugière, Lucas Moyet, Sophie Le Gall, Daniel Salvi, Marcel Kuntz, Marianne Tardif, Norbert Rolland

**Affiliations:** From the ‡University Grenoble Alpes, INRA, CNRS, CEA, IRIG-LPCV, 38000 Grenoble, France;; §University Grenoble Alpes, CEA, Inserm, IRIG-BGE, 38000 Grenoble, France

**Keywords:** Plant Biology*, Chloroplast, Cell fractionation*, Cellular organelles*, Subcellular analysis, Subcellular Separation, Chloroplast envelope

## Abstract

The chloroplast is a major plant cell organelle that fulfills essential metabolic and biosynthetic functions. Located at the interface between the chloroplast and other cell compartments, the chloroplast envelope system is a strategic barrier controlling the exchange of ions, metabolites and proteins, thus regulating essential metabolic functions (synthesis of hormones precursors, amino acids, pigments, sugars, vitamins, lipids, nucleotides etc.) of the plant cell. However, unraveling the contents of the chloroplast envelope proteome remains a difficult challenge; many proteins constituting this functional double membrane system remain to be identified. Indeed, the envelope contains only 1% of the chloroplast proteins (*i.e.* 0.4% of the whole cell proteome). In other words, most envelope proteins are so rare at the cell, chloroplast, or even envelope level, that they remained undetectable using targeted MS studies. Cross-contamination of chloroplast subcompartments by each other and by other cell compartments during cell fractionation, impedes accurate localization of many envelope proteins. The aim of the present study was to take advantage of technologically improved MS sensitivity to better define the proteome of the chloroplast envelope (differentiate genuine envelope proteins from contaminants). This MS-based analysis relied on an enrichment factor that was calculated for each protein identified in purified envelope fractions as compared with the value obtained for the same protein in crude cell extracts. Using this approach, a total of 1269 proteins were detected in purified envelope fractions, of which, 462 could be assigned an envelope localization by combining MS-based spectral count analyses with manual annotation using data from the literature and prediction tools. Many of such proteins being previously unknown envelope components, these data constitute a new resource of significant value to the broader plant science community aiming to define principles and molecular mechanisms controlling fundamental aspects of plastid biogenesis and functions.

## Abstract (precis)

By quantitatively comparing the proteomes of total leaf (crude cell extract) from Arabidopsis and purified chloroplast envelope fractions, this study makes available a novel parameter (calculated Enrichment Factor) for each putative envelope protein. This parameter provides important information to enable the more confident identification of genuine envelope components, distinguishing them from contaminants from other cellular/chloroplast compartments.

## Graphical Abstract

**Figure:**
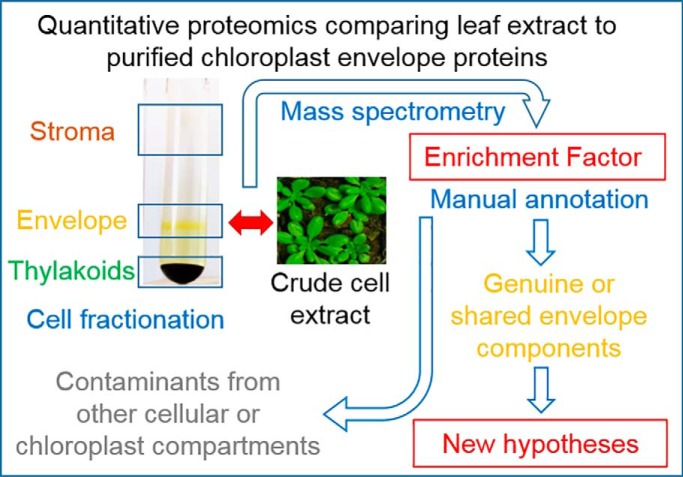

**Highlights**

- Identification of previously undetected chloroplast envelope proteins.
- Up to date manual annotation of genuine (or shared) envelope components.
- New hypotheses for localizations, functions, interactions among cell compartments.
- A new resource of significant value to the broader plant science community.

Understanding the functional diversification and evolution of cellular organelles requires the identification of their complete protein repertoires, which is the goal of organellar proteomics. Proteomics-based data, with analyses of nuclear genes that include predictions of subcellular location, is the method of choice to perform these analyses (1). Until now, it has not been technically feasible to generate an organelle fraction totally free from other cellular components. At the same time, increased mass spectrometry (MS) sensitivity allows detection of even trace impurities (2). The development of new software tools and the improvement of mass spectrometry technology have paved the way for a burst of MS data. Manual examination of these MS data is of great help to address the problem of contamination and to evaluate the validity of organelle proteome data. However, identification of a growing number of minor proteins with unknown (and sometimes unpredictable) subcellular localizations and functions still raises the question of the genuine subcellular localization of these uncharacterized proteins.

Plastids are major components of plant cells. They derive from a cyanobacterial ancestor that lost most of its genes after the establishment of endosymbiosis and eventually evolved as an organelle during evolution (3). Therefore, only one hundred of the three thousand different plastid proteins are still organelle-encoded; the remainder are encoded by nuclear genes (4). These ∼3000 proteins, encoded by such relocated genes, must be imported into chloroplast compartments following synthesis in the cytosol (5–7). Chloroplasts consist of several key sub-compartments including: (1) the envelope, a double membrane system surrounding the organelle and controlling the communication of the chloroplast with the rest of the cell (8), (2) the stroma, the soluble phase of the chloroplast and the main site for the conversion of carbon dioxide into carbohydrates, and *iii*) the thylakoid membrane, which is a widely organized internal membrane network where oxygenic photosynthesis takes place. Essential metabolic pathways occur, such as synthesis of lipids, pigments, amino acids, vitamins, starch, precursors of plant hormones, reduction of nitrite and sulfate, and photosynthesis occur in these subcompartments (9). Deciphering new regulatory mechanisms that control chloroplast dynamics and physiology, will generate important generic data on the regulation of these essential metabolic pathways in vascular plants. To that end, it is critical to characterize the chloroplast envelope proteome to obtain new information that will help define guidelines to better understand the chloroplast function and biogenesis.

Chloroplast envelope membrane proteins remain the most elusory part of the chloroplast proteome. Many envelope proteins remain to be characterized (known but uncharacterized envelope proteins) or identified (well-known envelope-associated functions carried out by yet unidentified proteins) (9). Indeed, because of their low abundance at the cell or even chloroplast scale (1–2% of the chloroplast proteins) (10), envelope proteins were historically, and remain much less understood than other plastid proteins. Subcellular and subplastidial proteomics, combining prior enrichment of subplastidial compartments, helped to decipher the basic membrane protein composition of the envelope proteomes of *Arabidopsis thaliana* and a few other plants (11–13). However, because of lower sensitivity of older MS hardware, studies targeting the envelope proteome did not rely on quantitative analyses by using crude cell extracts as a reference (see (12, 14)). Consequently, the quantitative parameter (enrichment factor or EF) resulting from the strong enrichment of the chloroplast envelope during subcellular and subplastidial fractionation was not available when analyzing MS data and annotating identified proteins. Further, contaminating proteins are often detected because they are among the most abundant proteins in other cellular compartments. Increasing knowledge about the subcellular localization of new proteins and the establishment of sophisticated proteome processing databases (*e.g.* MASCP Gator (15), SUBA (16) …) are expected to enhance and support the evaluation of chloroplast envelope proteome results.

The aim of the present study was to take advantage of new subcellular proteome databases (cited above), and improved mass spectrometry sensitivity to perform a quantitative proteome study comparing purified envelope fractions and crude cell extracts. We anticipated that these analyses would help *i*) identify specific envelope proteins with increasing accuracy *ii*) make available a novel parameter to clarify the subcellular or subplastidial localization of identified proteins and definitively exclude contaminants that are derived from other plastid or cell compartments. As expected, many more proteins were detected, including genuine but previously undetected chloroplast envelope proteins, but also many proteins deriving from other cell compartments. Accessibility to the quantitative approach enrichment factor (EF^1^ parameter), thanks to better MS sensitivity, allowed revisiting the chloroplast envelope proteome and to easily exclude most contaminants from other plastid or cell compartments.

## EXPERIMENTAL PROCEDURES

### 

#### 

##### Plant Material and Growth Conditions

*Arabidopsis thaliana* plants, Wassilewskija background (Ws), were grown in culture chambers at 23 °C (12-h light cycle) with a light intensity of 150 μmol/m^2^/s in standard conditions (17).

##### Purification of Arabidopsis Chloroplast and Its Subcompartments

All operations were carried out at 0–5 °C. For each envelope preparation, intact chloroplasts were obtained from 400–500 g of Arabidopsis (*Arabidopsis thaliana Ws*) leaves (*i.e.* 1800 5-week-old plants) and purified by isopyknic centrifugation using Percoll gradients (17). Note that all plants used for one preparation were grown at the same time. Purified intact chloroplasts were lysed in hypotonic medium containing protease inhibitors (10 mm MOPS-NaOH, pH 7.8, 4 mm MgCl_2_, 1 mm PMSF, 1 mm benzamidine, and 0.5 mm amino caproic acid), and envelope was purified from the lysate by centrifugation on sucrose gradients as previously described (17). To recover the envelope proteins, the yellow band of the sucrose gradient containing the envelope proteins was carefully aspirated with a pipette. Recovered envelope proteins were then diluted in 10 mm MOPS-NaOH pH 7.8 buffer (containing protease inhibitors: 1 mm PMSF, 1 mm benzamidine, and 0.5 mm α-aminocaproic acid), and pelleted by centrifugation at 110,000 *g* for 1 h (Beckman SW 41 Ti rotor) (17). Envelope proteins were diluted in 100 μl of the same medium containing protease inhibitors (1 mm PMSF, 1 mm benzamidine, and 0.5 mm α-aminocaproic acid) and stored in liquid nitrogen (an average of ∼100 μg of envelope proteins was obtained from each preparation).

##### Extraction of Crude Leaf Extract (CCE)

All operations were carried out at 0–5 °C. Total leaf proteins were obtained from three leaves of three independent Arabidopsis (*Arabidopsis thaliana Ws*) plantlets. Leaf material was homogenized in 200 μl of extraction buffer (30 mm tetrasodium pyrophosphate, 50 mm Tris pH 6.8, SDS 1% [v/v]) and then centrifuged at 16,000 × *g* for 5 min. Crude leaf extract (CE) was then recovered by carefully aspirating the supernatant. Total protein concentration was measured using the Bradford dye-binding method (Bio-Rad protein assay). Because of the risk of proteolysis in crude cell extracts, these protein samples were not stored in liquid nitrogen, but separated on SDS-PAGE for further MS analyses immediately after preparation.

##### Purification of Arabidopsis Membrane Fractions Enriched in Microsomal and Tonoplast Membranes

Fractions enriched in microsomal and tonoplast membranes from *Arabidopsis* plants were purified according to a previously described protocol (18). All operations were carried out at 0–5 °C. Thirteen grams of Arabidopsis (*Arabidopsis thaliana* Ws) leaves from 4 week old plants was used as starting material. These leaves were first incubated in 25 ml of grinding buffer (0.3 m mannitol; 20 mm MOPS; 2 mm EGTA; 0.5% (w/v) polyvinylpyrrolidone 25; 0.5% (w/v) BSA (Bovine Serum Albumin); 5 mm DTT; 1 mm PMSF; 5 mm α-aminocaproic acid; 1 mm benzamidine; pH 8.0). Leaves were then ground using a pestle in a mortar containing 10 ml of micro silica sand from Fontainebleau. After addition of 200 ml of grinding buffer, the suspension was centrifuged for 10 min at 2200 × *g* and the supernatant was stored for further purification steps. Then, light membrane vesicles (microsomes) were enriched from this supernatant following 4 successive centrifugation steps: 2 centrifugations of 5 min at 9500 × *g* and 1 centrifugation of 15 min at 25,000 × *g*. The resulting supernatant was then centrifuged for 45 min at 100,000 × *g*. The resulting pellet (microsomal membranes) was then stored on ice. This pellet was then diluted in 2 ml of washing buffer (0.3 m Mannitol; 10 mm MOPS; 1 mm EDTA; 1 mm PMSF; 5 mm α-aminocaproic acid; 1 mm benzamidine; pH 7.4) and loaded on the top of a discontinuous gradient made of 4 layers of sucrose (25% (w/v); 30% (w/v); 38% (w/v); 45% (w/v) sucrose in 50 mm MOPS; 2 mm EGTA; 2 mm PMSF; 10 mm α-aminocaproic acid; 2 mm benzamidine; pH 7.4). After centrifugation for 4 h at 80,000 × *g*, the tonoplast enriched fraction was collected at the interface of the 25 and 30% gradient layers. To remove sucrose, this fraction was then diluted 4 times in washing buffer lacking mannitol and then centrifuged for 1 h at 175,000 × *g*. The resulting pellet (tonoplast) was then diluted in a minimal volume of washing buffer lacking mannitol and stored at −80 °C until use.

##### SDS-PAGE and Western Blotting Analyses

SDS-PAGE analyses were performed as described by Chua (19). For Western blotting analyses, gels were transferred to a nitrocellulose membrane (BA85, Schleicher & Schuell). Various markers of cell compartments were detected using several antibodies (in-house, commercially available, or gifts from colleagues): GFP (GFP-2A5, obtained from Euromedex, 67458 Mundolsheim, France), HMA1 (20) as an envelope marker, LHCP (provided by Dr. Olivier Vallon, IBPC Paris) as a thylakoid marker, KARI (provided by Dr. Renaud Dumas, LPCV Grenoble) as a marker from the stroma, Bip (obtained from Agrisera) as a marker from the ER, V-ATPase (21) as a tonoplast marker, bobTIP (provided by Prof. Nathalie Leborgne-Castel (INRA/Université de Bourgogne) directed against tonoplast TIP1.1, and FtsY (provided by Dr. Laurent Nussaume, CEA Cadarache) directed against chloroplast FtsY.

##### Experimental Design

Total extracts and envelope fractions were prepared in triplicate. For envelope sample preparation, triplicates were treated as technical replicates because the potential biological variability is already averaged across the 1800 5-week-old plants that were needed to obtain a single sample of purified envelope fraction. Total leaf proteins were extracted from three leaves of three independent *Arabidopsis* plantlets that were used to further extract envelope fractions.

##### Proteins Digestion for Proteomics Analyses

Each protein sample (10 μg) was stacked by a 1 cm-migration on the top of a NuPAGE 4–12% gel, Invitrogen) before Coomassie blue staining (R250, Bio-Rad). Gel bands of concentrated proteins were manually excised and cut in pieces before being washed by 6 successive incubations of 15 min in 25 mm NH_4_HCO_3_ containing 50% (v/v) acetonitrile. Gel pieces were then dehydrated in 100% acetonitrile and incubated at 53 °C with 10 mm DTT in 25 mm NH_4_HCO_3_ for 45 min and in the dark with 55 mm iodoacetamide in 25 mm NH_4_HCO_3_ for 35 min. Alkylation was stopped by adding 10 mm DTT in 25 mm NH_4_HCO_3_ and mixing for 10 min. Gel pieces were then washed again by incubation in 25 mm NH_4_HCO_3_ before dehydration with 100% acetonitrile. Modified trypsin (Promega, sequencing grade) in 25 mm NH_4_HCO_3_ was added to the dehydrated gel pieces for an overnight incubation at 37 °C. Peptides were then extracted from gel pieces in three 15-min sequential extraction steps in 30 μl of 50% acetonitrile, 30 μl of 5% formic acid and finally 30 μl of 100% acetonitrile. The pooled supernatants were then vacuum-dried.

##### Nano-LC-MS/MS Analyses

The dried extracted peptides were resuspended in 5% acetonitrile and 0.1% trifluoroacetic acid and analyzed by online nanoLC-MS/MS (NCS, and Q-Ex_HF, Thermo Fischer Scientific). Peptides were sampled on a 300 μm × 5 mm PepMap C18 precolumn and separated on a reprosyl 25 cm 1.9 μm (Cluzeau). The nanoLC method used a 140-min gradient ranging from 4% to 40% acetronitrile in 0.1% formic acid (in 123 min) and wash to 90% and equilibration at 4% at a flow rate of 300 nL/min. MS and MS/MS data were acquired using Xcalibur (Thermo Fischer Scientific). Spray voltage and heated capillary were set at 2 kV and 270 °C, respectively. Survey full-scan MS spectra (*m*/*z* = 400–1600) were acquired in the Orbitrap with a resolution of 60,000 after accumulation of 10^6^ ions (maximum filling time: 200 ms). The 20 most intense ions from the preview survey scan delivered by the Orbitrap were fragmented by collision induced dissociation (collision energy 30%) in the LTQ after accumulation of 10^5^ ions (maximum filling time: 50 ms).

##### Database Searches and Results Processing of MS Data

Peak lists files were generated using Mascot Daemon. MS/MS spectra were searched using Mascot 2.6.0 (Matrix Science) against the target-decoy version of a compilation of the *A. thaliana* protein database (nuclear, mitochondrial and plastid genome; TAIR v10.0; December 14, 2010; 35,386 entries) and a home-made list of contaminants, frequently observed in proteomics analyses (249 entries). Trypsin/P was chosen as the enzyme with a maximum of 2 missed cleavages allowed. Precursor and fragment mass error tolerances were set at 10 ppm and 0.025 Da, respectively. Peptide modifications allowed during the search were: carbamidomethyl (C, fixed) acetyl (Protein N-term, variable) and oxidation (M, variable).

The Proline software (http://proline.profiproteomics.fr/) was used to filter the results (filters at the replicate level were conservation of only rank 1 peptides, peptide identification FDR < 1% as calculated on peptide scores by employing the reverse database strategy, minimum peptide score of 25, peptide length ≥ 7, and minimum of 1 specific peptide per identified protein group. Next identifications were merged over the whole experiment and the filter requiring a minimum of 1 specific peptide per protein group was applied again. Spectral count (SC), specific spectral count (SSC) and weighted spectral count (WSC, calculated as described in (22)) were computed within each replicate. The identifications resulting from this validation pipeline are also available as excel tables (see supplemental Table S1).

Additional filters were applied manually: (1) contaminants (keratin, … ) were discarded, (2) protein groups detected with only one specific peptide were partially filtered by excluding proteins having a WSC = 1 over the whole experiment. In other words, proteins not detected in a fraction (SWSC = 0) but detected in a different fraction with only SWSC = 1 were discarded from the analysis. The WSC values were normalized by the total WSC within each replicate. The ratio of the sum of normalized WSC over triplicates (WSC_Env/WSC_CCE) was used as an estimator of the enrichment of each protein in the envelope fraction compared with the total extract. This ratio could be easily established for abundant envelope proteins that were detected in both envelope and crude cell extracts. However, some poorly abundant envelope proteins were not detected in crude cell extract replicates (*i.e.* WSC_CCE = 0). To estimate their enrichment factor, their spectral count values were thus modified, and a spectral count of 1 spectrum was added to both norm_WSC_CCE and norm_WSC_Env, as previously described (24).

Boxplots were generated by the R-studio software using the function boxplot: boxplot(log(EF)∼local,las = 2,ylab = “log(EF)”,cex.axis = 0.8,col = “gray”). The Max and the min of boxplots were defined automatically by the software according to the values of the data provided in the form of a matrix (table) at the beginning of the script.

##### Construction of Vectors for Stable Expression in Arabidopsis

To construct the vectors for FtsY::GFP, UP1::GFP, VTE1::GFP and eIF-5A::GFP overexpression in Arabidopsis, the coding region of Arabidopsis correspondent protein was PCR-amplified using the two flanking primers SalI-*N*-ter and NcoI-*C*-ter (see supplemental Table S2). The amplified fragments were first inserted by cloning a blunt-ended fragment in an intermediate plasmid, the plasmid pBluescript (pKS), and then sequenced to rule out the possibility of PCR-induced mutations. The SalI-NcoI fragment cleaved from each plasmid was inserted into the SalI-NcoI digested GFP reporter plasmid (puc19–35Ω-sGFP(S65T)) (25). The plasmid was digested with HindIII and *EcoR*I to isolate the inserts encoding both the protein of interest and the GFP fusion protein. This fragment was subsequently introduced into the HindIII-*EcoR*I-digested pEL103 binary vector (kan resistance to transform wild-type plants). SFR2::CFP and TSP9::CFP were constructed by recombination using the Gateway recombination system and the ^35^S-CFP construction.

##### Arabidopsis Transformation and Confocal Microscopy Analyses

Wild-type Arabidopsis plants ecotype WS were transformed by dipping floral buds of 4-week-old plants into an *Agrobacterium tumefaciens* (C58 strain) solution containing a surfactant (Silwet l-77) according to Clough and Bent (26). Primary transformants were selected on Murashige and Skoog medium (Murashige and Skoog MS5519, 1% [w/v] sucrose, and 1% [w/v] agarose) containing 100 mg/l kanamycin. Only lines segregating 3:1 for the resistance to kanamycin and expressing the recombinant protein were selected for further analyses (subcellular localization of proteins using confocal microscopy). Fluorescence microscopy was then performed with a confocal laser-scanning microscope (TCS-SP2, Leica, Deerfield, IL).

## RESULTS

### 

#### 

##### Toward a Deep-rooted and Reliable Investigation of the Chloroplast Envelope Proteome: Overview of the Complete Strategy

**Fig. 1.**
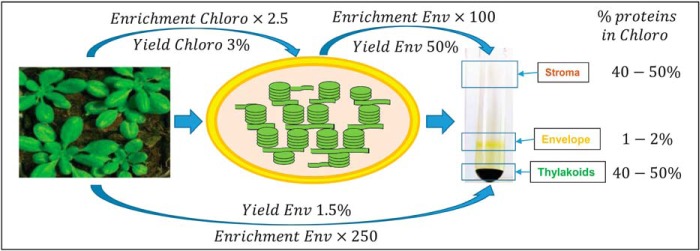
**Theoretical enrichment factor (EF) of envelope proteins, from crude cell extract to purified envelope fractions.** Chloroplasts contain 40% of plant cell proteins. Thus, an enrichment factor of chloroplast proteins in Percoll-purified chloroplast fraction compared with total extract would be 2.5. Because the chloroplast envelope represents a minor chloroplast component (*i.e.* only 1–2% of the chloroplast proteins), envelope proteins should be around 250 times more abundant in the purified envelope fraction when compared with whole cell extract. This enrichment factor (EF) could be used to discriminate genuine envelope proteins from contaminants.

Chloroplast proteins are major components of plant cells, representing 40% of total cellular protein. Chloroplasts are surrounded by two envelope membranes (the chloroplast envelope) that contain only 1–2% of all chloroplast proteins. Therefore, chloroplast envelope proteins represent less than 1/250 of the total cellular proteins (Fig. 1). Thus, when comparing the compositions of crude leaf extract and purified envelope vesicles, a high theoretical enrichment factor (EF) of specific envelope proteins is expected.

Sensitivity of MS techniques have been greatly improved during the last decade. Although only 100 proteins (including contaminants from other chloroplast sub-compartments) could be identified in purified chloroplast envelope fractions 15 years ago (27), 700 proteins were detected in 2010 (12). Today, thanks to the continuous improvement of MS techniques and instruments, we are able to detect more components in a complex sample, to generate quantitative data, and to statistically validate the above-cited enrichment factors. Thus, the aim of the present study was to analyze crude cell extracts and envelope fractions using a MS-based approach to (1) detect more components in chloroplast envelope samples, and (2) differentiate genuine (highly enriched) chloroplast envelope proteins from components derived from other cell or plastid compartments by comparing quantitative data of each protein in both crude cell extracts and envelope fractions.

Crude cell extracts and envelope fractions were prepared in independent triplicates. Proteins (15 μg) in each sample were separated by 12% SDS-PAGE gel with Coomassie blue staining to assess the reproducibility of the preparation and homogeneity of the samples (see supplemental Fig. S1). As expected, similar protein profiles were observed over triplicates. Well-known abundant markers associated with the chloroplast were revealed in their appropriate fractions. Indeed, the large subunit of Rubisco (RbcL) and the light harvesting complex protein (LhcP) were highly and proportionally detected in all three total cell extracts (supplemental Fig. S1*A*, lanes CCE1–3). Phosphate/triose-phosphate transporter (TPT), the major protein of the chloroplast envelope sub-fraction, was largely enriched in each purified envelope fraction (supplemental Fig. S1*B*, lanes E1–3). These triplicate biological samples were then analyzed by MS, after concentration of the protein samples (10 μg) in the top of a NuPage 4–12% (supplemental Fig. S1*C*).

##### Crude Data Analysis: Distribution and Predicted Subcellular Classification

Almost 3000 non-redundant proteins (2948) were identified from our automatic validation treatment of the six analyses performed (supplemental Table S1). An additional filter, which consisted of eliminating proteins that were absent in a fraction (SWSC = 0) and detected in the other fraction with only SWSC = 1, was applied to i) reduce the risk of false identifications among proteins identified with only one specific peptide ii) prevent any Enrichment Factor from being based on a single spectral count. This filter eliminated 108 proteins out of 366 initially identified only in the envelope fraction (30%) and 360 proteins out of 1571 proteins initially identified only in the total extract fraction (23%) (compare supplemental Table S1 with supplemental Table S3 or S5).

We obtained a final set of 2480 Arabidopsis proteins (supplemental Table S3) that resulted from the cross of 2222 proteins identified in the crude cell extracts (minimum of 1 specific peptide in the CCE triplicate) and 1269 proteins identified in envelope fractions (minimum of 1 specific peptide in the Env triplicate). In the current work, proteins present in purified chloroplast envelope fractions were detected at an unprecedentedly high level of sensitivity. Indeed, ca 700 proteins were previously identified in comparable envelope fractions using the same number of biological samples (12).

**Fig. 2.**
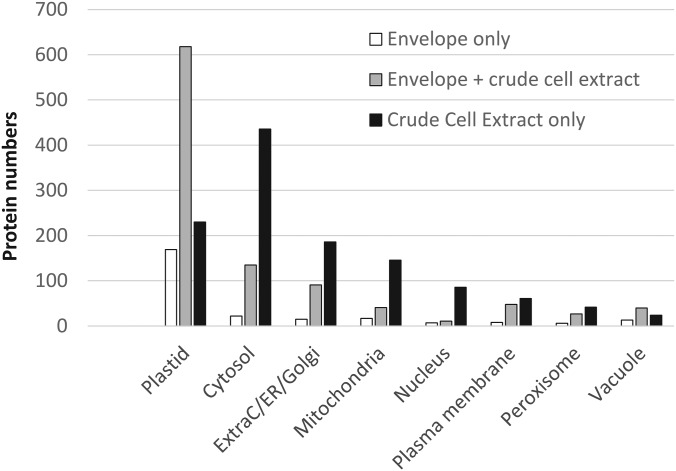
**Predicted subcellular localization of proteins identified in purified envelope fractions and crude cell extracts according to the SUBA3 database (SUBAcon, see (88).** Note that only 1 of the almost 2500 proteins detected during this work was not present in the SUBA3 database. Proteins identified in envelope fractions are enriched in predicted plastid proteins whereas crude cell extracts (CCE) contain more proteins predicted to be localized in other cell compartments. Envelope only (white): proteins only detected in the envelope fraction. Envelope + CCE (gray): proteins detected in both envelope and crude cell extract. CCE only (black): proteins detected in crude cell extract and absent from the list of envelope proteins.

As a preliminary analysis, subcellular localizations of all detected proteins were predicted using information (consensus localization) available in the SUBA3 database (see supplemental Table S3). According to this analysis, 1017 proteins (41% of the 2480 detected proteins) were assigned to the plastid. More than half (618 proteins, 61%) of these plastid proteins were shared by envelope and crude extract lists (Fig. 2). These shared plastid proteins might represent major envelope proteins or abundant plastid proteins (stroma/thylakoids) cross-contaminating the purified envelope fractions. Part (169 proteins, 17%) of these plastid proteins were only detected in envelope fractions; they are expected to represent minor envelope proteins that are too scarce to be detected in crude cell extracts. Altogether, 77% (787 proteins) of the plastid proteins were identified in the envelope fraction. On the other hand, 23% (230 proteins) of the plastid proteins were only detected in crude cell extracts. These last proteins are anticipated to be stroma or thylakoid proteins that are abundant enough to be detected in crude cell extracts, but too scarce to be detected in the list of stroma or thylakoid proteins that commonly contaminate purified envelope fractions.

Beyond plastid proteins, many identified proteins were predicted to be components derived from other cell compartments (cytosol, mitochondria, nucleus, plasma membrane, etc.). This MS/MS analysis identified 593 proteins from the cytosol, 292 from the secretory system, 204 from the mitochondria, 104 from the nucleus, 117 from the plasma membrane, 75 from the peroxisome, and 77 from the vacuole (see supplemental Table S4). Interestingly, out of these 593 cytosolic proteins, 74% of them (436 proteins, see supplemental Table S4), were only detected in the crude cell extract, whereas only 4% (22 proteins) were detected exclusively in the envelope fractions. The same is true for other cell compartments, because most ExtraC/ER/golgi (64%), mitochondria (72%), nucleus (83%), plasma membrane (52%) and peroxisomal (56%) proteins were only detected in crude cell extracts (see supplemental Table S4). Surprisingly, only vacuolar proteins show a more balanced repartition between envelope fractions and crude cell extracts; 31% of them being only detected in crude cell extracts and 17% of them being only detected in envelope fractions, thus suggesting that cross-contamination of the envelope preparations with vacuolar proteins is more pronounced (see supplemental Table S4 and Fig. 2).

##### Annotation of the 1269 Proteins Detected in Purified Envelope Fractions

Because our objective was to concentrate on the chloroplast envelope proteome, we targeted our analysis toward the 1269 proteins that were detected at least once in purified envelope fractions (see supplemental Table S5).

To explore these data, we then performed a manual annotation of their subcellular and subplastidial localization, accurate description and function (supplemental Fig. S2). To do so, information about protein description, function, and subcellular localization were sequentially and manually searched in several public databases (TAIR, Uniprot, NCBI, MAPMAN…) and the appropriate literature (PubMed, searching for either AGI numbers or protein names…). Then, the 1269 proteins were tested for the presence of a predictable chloroplast transit peptide using ChloroP (28) and TargetP (29) prediction tools. However, it is important to note here that outer membrane proteins do not typically possess a predictable transit peptide; the same is true for the few chloroplast-encoded proteins. Further, as shown in supplemental Fig. S2, two reliable and effective databases termed “SUBA3” (30) and “MASCP Gator” (31) were used to determine if some proteins are known components of other cell compartments (mitochondria, plasma membrane, cytosol…). Indeed, both “SUBA3” and “MASCP Gator” provided substantial information by indicating whether each protein has previously been detected by MS in specific cell compartments, if its subcellular localization was determined using *in planta* expression of GFP-tagged forms, and if a consensus subcellular localization can be deduced from all available subcellular prediction tools. Finally, present data were also overlapped with the list of proteins, previously identified in the envelope fractions, and their subplastidial localizations present in the AT_CHLORO database (12) (see supplemental Table S6).

We first considered previous MS-based detection of each protein in specific cell compartments (using MASCP-Gator and SUBA3) as a key criterion (supplemental Fig. S2). For example, when a protein was only and repeatedly detected in the plastid and never or rarely in another cell compartment, association of this protein to the plastid compartment was considered as highly plausible (even if this protein was lacking a classical and predictable chloroplast transit peptide). On the contrary, when a protein was predicted to contain a chloroplast transit peptide but repeatedly detected in mitochondria or any other cell compartment, it was excluded from the list of plastid proteins.

##### Validation of the EF Value to Predict the Subcellular and Subplastidial Localization of the 1269 Proteins Detected in Purified Envelope Fractions

To further support the known or predicted localization of the 1269 identified proteins, spectral count (SC), specific spectral count (SSC) and weighted spectral count (WSC) were computed for each detected protein within each replicate. The WSC of each sample (crude extract or envelope) was normalized independently using the total WSC of each replicate related to the sum of one replicate (supplemental Table S2). A ratio (EF = Enrichment Factor) of the sum of normalized WSC over each triplicate (EF = norm_WSC_Env/norm_WSC_CCE) was calculated and tentatively used as an estimator of the enrichment of each protein in the envelope fraction compared with the crude cell extract. When proteins were only detected in envelope fractions (*i.e.* norm_WSC_CCE = 0), one spectral count (+1) was added to both norm_WSC_Env and norm_WSC_CCE to calculate this ratio (see supplemental Table S5, column EF estimator) as previously described (24).

To test the reliability of this EF parameter, we first created a “safe set” of proteins. These proteins were selected as follows: (1) their subcellular localizations were experimentally determined *in planta* using GFP-fusion experiments and confocal microscopy. Alternatively, 42 chloroplast- and 2 mitochondria-encoded proteins were considered as genuine proteins from their respective organelles, (2) SUBAcon, *i.e.* the consensus of all localization prediction tools present in the SUBA3 database, supports the data issued from the *in planta* experiments, (3) the subplastidial localization of the proteins was previously (12) and unambiguously determined (thus excluding proteins only detected during the present work). Combining the above-cited parameters, only 175 proteins could be selected, representing 14% of the 1269 proteins detected in the purified envelope fractions. Interestingly, this set of 175 proteins contains protein groups from the whole cell and the chloroplast compartments (see supplemental Fig. S3). In good agreement with their expected enrichment in purified envelope fractions compared with crude cell extracts (see Fig. 1), envelope proteins (inner or outer membrane) proteins from the “safe set” were highly enriched (EF > 10) in purified envelope fractions (see supplemental Fig. S3). This enrichment was less pronounced for envelope proteins previously shown to be shared between envelope and other plastid compartments (stroma and thylakoids). Interestingly, if one excludes proteins from the vacuole, all other cell- and plastid-associated components showed an average EF value far below (< 1) the value obtained for genuine envelope proteins.

Having confirmed the correlation between genuine envelope localization and effective enrichment (high EF values), we then used (when required) this parameter to help predicting the subcellular and subplastidial localization of the “Negative of the safe set,” *i.e.* the 1094 proteins whose localization could not be unambiguously deduced from previous analyses (see bottom of supplemental Fig. S2). We especially considered this EF value as a crucial parameter to classify proteins as genuine envelope proteins (*i.e.* EF value above 5, highly enriched in the purified envelope fraction when compared with crude cell extract). When the EF value was lower (*i.e.* 1 < EF < 5) for an envelope protein, data from AT_CHLORO were essential to discriminate proteins, shared by envelope and other plastidial compartments (stroma or thylakoids), from proteins that were probably contaminating (very low EF value, *i.e.* EF < 1) the purified envelope fractions. Finally, we combined rare data from the literature and useful data recently published by Schleiff and colleagues (14) to classify the proteins as either inner or outer envelope proteins (see supplemental Table S5, column “Simm *et al.*, 2014”).

**Fig. 3.**
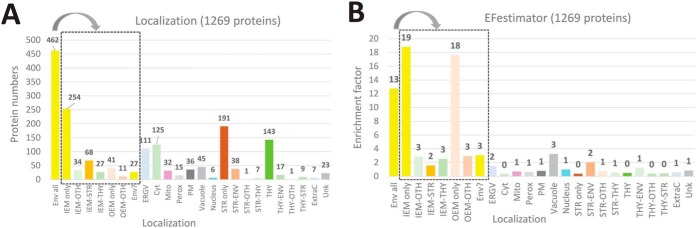
**Subplastidial and subcellular localizations of the 1269 proteins detected in purified envelope fractions as deduced from manual annotation (see supplemental Table S5).** The black square contains subcategories of envelope proteins (Env All): proteins only detected in envelope (IEM or OEM only), proteins shared with other undefined plastid compartments (IEM- or OEM-OTH), stroma (IEM-STR) or thylakoid (IEM-THY) or envelope candidates (Env?). *A*, As deduced from information present in the literature, databases and prediction tools (see Fig. 3), only 462 (383 Env + 52 OEM + 27 Env?) of the 1269 proteins are predicted to be associated with envelope membranes. *B*, As expected (see Fig. 1 and supplemental Fig. S3, “safe set”), the average enrichment factor of predicted envelope proteins is far above that of proteins associated with other plastid or cell compartments. Note the relatively low EF values of proteins shared between envelope and other plastid compartments, and the surprisingly high EF value of vacuolar proteins. IEM; inner envelope membrane, OEM; outer envelope membrane, Env?; Envelope candidates; ERGV: endoplasmic reticulum/Golgi, Cyto; cytosol, Mito; mitochondria, Perox; peroxisome, PM; plasma membrane, Vacuole; vacuole, Nucleus; nucleus, Str; stroma, thy; thylakoid, Ext; extracellular localization, Unk; unknown and unpredictable localization, OTH; other undefined localization.

Combining all information present in existing databases and literature, and by following the strategy depicted in supplemental Fig. S2, we were able to classify all identified proteins in various categories including their subcellular and subplastidial localizations, specific activities, and main functions (see supplemental Table S5, columns “Curated function” and “Curated description”). As depicted in Fig. 3 *A*, out of the 1269 proteins that were detected at least once in purified envelope fractions, 462 proteins (36%) could be classified as envelope proteins (see Fig. 3*A*, Env-All). This includes 383 (30%) inner envelope proteins (see Fig. 3*A*, IEM), 52 (4%) outer envelope proteins (see Fig. 3*A*, OEM) and 27 (2%) envelope candidates (see Fig. 3*A*, Env?). These envelope candidates were mostly uncharacterized proteins that are never or rarely detected in any other cell compartments, or enriched in the envelope and contain a predicted cTP…). Out of the 462 above-cited envelope proteins (Fig. 3*A*, Env-All), 254 inner and 41 outer envelope proteins (see Fig. 3*A*, IEM and OEM only) were highly enriched (or were only detected) in the purified envelope fractions (see Fig. 3*B*). The other 130 are probably envelope components that are shared with other compartments (see Fig. 3*A*, IEM-stroma, IEM-thylakoids, IEM-other, OEM-other) were less enriched (see Fig. 3*B*).

However, because of highly improved detection sensitivity of current spectrometers, we not only revealed several minor envelope proteins, but also many contaminants from other chloroplast and cell compartments. As expected from previous studies, the main contaminants (Fig. 3*A*) were derived from the stroma (237 proteins, *i.e.* 191 + 38 + 1 + 7) and thylakoids (170 proteins, *i.e.* 143 + 17 + 1 + 9). However, being well-known or predicted plastid components, these proteins were easily excluded from the envelope list because of their very low EF values (EF < 0.5; see supplemental Table S5, and Fig. 3*B*).

All other identified proteins are well-known (abundant markers) or expected contaminants deriving from other cell compartments. Many (10%, 125 proteins) are derived from the cytosol. Most of these are linked to cytosolic translation of proteins (ribosomal proteins) with an average EF value of 0.3. An equivalent number (9%, 111 proteins) are either ER/Golgi proteins or additional cytosolic proteins linked to intracellular trafficking functions (average EF value of 1.5). 45 proteins (4%) are vacuolar components with a surprisingly non-negligible average EF value of 3.3 (see Fig. 3*B*). 36 proteins (3%) were assigned to the plasma membrane (average EF value of 0.8). 32 proteins (3%) were assigned to the mitochondria (average EF value of 0.7). Six proteins (<1%) were assigned to the nucleus (average EF value of 1.0). 7 proteins (< 1%) were assigned as extracellular (average EF value of 0.6). Finally, it was not possible to identify or predict a subcellular localization for 23 proteins (2%). According to this classification, the average EF for the 462 envelope proteins (see Fig. 3*B*, Env-All) was 13, whereas most other categories were given a value around 10 times lower (see Fig. 3*B*). The only exceptions were the 27 envelope candidates (see Fig. 3*B*, Env?) with an average EF value of 3, and the 45 vacuolar components (see Fig. 3*B*, vacuole) with the same average EF value of 3.

Altogether, following the strategy depicted in supplemental Fig. S2, a total of 869 (462 env + 237 stroma + 170 thylakoids) proteins (69%) were classified as well-known or good candidates for true chloroplast localization. Only 53% of these chloroplast proteins were predicted to be envelope components.

##### Comparison to Recent Studies Targeting the Chloroplast Envelope Proteome

##### Previously Undetected Proteins in Any MS/MS-based Study

We first compared proteins detected in purified envelope fractions with all previous proteomics studies referenced in MASCP-Gator and SUBA3. According to these databases, 70 proteins (of the 1269 proteins identified in envelope fractions) were detected for the first time using a MS-based approach (see supplemental Table S5, proteins classified as “nd” in column “Experimental evidence”). In good agreement with the targeted subcellular fraction, and with improved sensitivity of our MS-based approach, most (53) of these previously undetected 70 proteins were known or predicted envelope components (29 IEM, 9 OEM, 15 Env?), whereas less (17) were contaminants deriving either from ER or Golgi (5), thylakoids (4), cytosol (3), stroma (2), mitochondria (2), or plasma membrane (1). No contaminants came from the peroxisome, the nucleus, the vacuole or the extracellular compartment. In other words, because most other cell compartments were deeply analyzed in previous targeted MS-based studies, a large fraction of proteins, present in other cell compartments and detected during this work, were previously detected using targeted MS. One might even be skeptical about the classification (subcellular localization) of the very few (17) above-cited non-envelope proteins knowing that the probability of detecting them in a purified envelope fraction is lower than the probability of detecting them in the cell compartments where they actually reside.

##### Previously Undetected Proteins in MS/MS-based Study Targeting Envelope Fractions

Because one of the main goals of the present study was to obtain a better understanding of the composition of the chloroplast envelope, the present data were also overlapped with our previous data stored in AT_CHLORO (12). Out of the 1269 proteins identified in the purified envelope fractions during the present study, 433 proteins were not previously detected in any chloroplast sub-compartment (envelope, stroma, thylakoid) in our previous work (12) (see supplemental Table S5, column “Env/Str/Thy, AT_CHLORO”). Out of these 433 newly detected proteins, 87 could be assigned an envelope localization (including 24 envelope candidates, *i.e.* “Env?”); 98 were classified as ER/Golgi components; 90 as “cytosol”; 29 as “mitochondria”; 22 as “thylakoid”; 29 as “plasma membrane”, 29 as “vacuole”; 12 as “stroma”; 5 as “extracellular compartment”; 5 as “nucleus”; 5 as “peroxisome” and 22 had a subcellular localization that could not be proposed (see supplemental Table S5, Column Simplified location this work, category “unknown”). By using prediction tools (see Fig. 2) and taking advantage of better MS detection, we not only detected several new minor envelope proteins, but also many other contaminants deriving from other chloroplast and cell compartments. Indeed, as shown in supplemental Fig. S4*B*, the present study provided a better overview of the composition of the chloroplast envelope (92 proteins) when compared with our previous work (12).

On the other hand, 91 proteins, present in AT_CHLORO (12), were not detected in the purified envelope fractions during the present work (see supplemental Table S6 and supplemental Fig. S4*C*). Out of these 91 proteins, only 16 were associated to the chloroplast envelope whereas most (75) of the other proteins were contaminants from other compartments. It is also important to note here that part (38 proteins) of these 91 proteins, previously detected by Ferro *et al.* (12), were also detected during this work, but only in crude cell extracts (see supplemental Table S6). This finding is in good agreement with their known or predicted subcellular localization (13 thylakoids, 12 cytosol, 6 stroma, 3 mitochondria, 2 peroxisome, 1 plasma membrane, 1 envelope candidate).

We next compared our results to a more recent study by Simm and colleagues (14) who aimed to identify the core proteome of the chloroplast envelope by analyzing the envelope proteome of three plant species. As shown in supplemental Fig. S4*B* and supplemental Table S5, 98 (*i.e.* 90 + 8 in supplemental Fig. S4*C*) proteins were classified as envelope components in both studies. Only 90 proteins were detected in all three studies. Many proteins (116, *i.e.* 82 + 34 in supplemental Fig. S4*C*) identified during the study of Simm and colleagues (14) were detected during the present work, but assigned other subcellular or subplastidial localizations (82 for thylakoids, 14 for stroma and a few components from the mitochondria, peroxisome, ER/Golgi, plasma membrane and cytosol). Finally, 57 proteins (*i.e.* 54 + 3 in supplemental Fig. S4*C*), detected during the study of Simm and colleagues (14), were not detected here. Only one protein was classified as an envelope component (14) whereas most others were classified as “unknown,” “thylakoid,” “plastid,” or “stroma.”

To conclude, when considering data in supplemental Fig. S4, it appears that our new approach provides a better overview of the chloroplast envelope composition, when compared with earlier, but relatively recent studies (12, 14). To cite a few examples (see Table I), a novel isoform of the solute conducting channel OEP24 (32), termed OEP24A (At1g45170) was identified during this work as an outer envelope pore protein (see supplemental Table S5, and Table I). This protein, previously detected by Simm *et al.* (14), was not present in the AT_CHLORO database. Two components of the TOC translocon, annotated TOC90 (At5g20300) (33) and TOC120 (At3g16620) (34) were detected during the present work but not detected during our previous study (12). Only TOC120 was previously detected during the study performed by Simm *et al.* (14) (see supplemental Table S5). SP1 (At1g63900), a chloroplast outer membrane E3 ligase controlling protein import (35), which was not detected in previous proteomic studies, was also detected during the present work (see supplemental Table S5). Additionally, we (see Table I) identified TIC20-I (At1g04940) and TIC20-V (At5g55710), which are involved in protein precursor import into chloroplasts at the inner envelope membrane (36, 37). TIC20-I, which was recently discovered to specifically interact with photosynthesis-related pre-proteins. It was found to be partially redundant with TIC20-IV, but not with TIC20-II or TIC20-V (36, 37). This protein was not identified by either earlier proteomic studies (12, 14) but was detected in the present work (see Table I).

**Table I:** Detection of representative inner and outer envelope membrane proteins. Protein accessions are provided (AGI numbers) together with their short names (Name). Detection (X) of these proteins in the present work, in the work published by Simm et al. (Core proteome, see 14), or by Ferro et al. (AT_CHLORO database, see 12) is indicated. The enrichment factor (EF) of each protein in the envelope fraction compared to the crude cell extract is indicated in the EF estimator column. na, means that the protein was detected in the whole chloroplast fraction but not in the purified envelope fractions. Bold accessions are known envelope proteins that were not detected in previous studies targeting the envelope proteome

AGI	Name	This work (2019)	Core proteome (2014)	AT_CHLORO database (2010)	EF estimator (this work)
**AT1G04940**	**TIC20-I**	X	–	–	10.8
**AT2G47840**	**TIC20-II**	X	–	–	7
AT5G55710	TIC20-V	X	–	na	0.7
AT3G23710	TIC22-III	X	–	X	10.9
AT4G33350	TIC22-IV	X	X	X	12
AT4G23430	TIC32-IVa	X	–	X	10
AT5G16620	TIC40	X	X	X	17.4
AT2G24820	TIC55-II	X	X	X	8.8
AT4G25650	TIC55-IV	X	X	X	94.3
ATCG01130	TIC214	X	X	X	41.8
**AT2G25660**	**TIC236**	X	–	–	15.9
AT5G22640	TIC100	X	X	X	90.2
AT1G06950	TIC110	X	X	X	10
**AT5G20300**	**TOC90**	X	–	–	8.9
AT3G16620	TOC120	X	X	–	5.2
**AT1G63900**	**SP1**	X	–	–	8.0
**AT1G21650**	**SECA2**	X	–	–	27.8
AT2G31530	SCY2	X	–	X	7.9
AT1G45170	OEP24A	X	X	–	1.9
AT3G52230	OEP24	X	X	X	4.1
AT5G42960	OEP24-II	X	–	X	1.9

##### Functional Classification of the 1269 Proteins Identified in Purified Envelope Fractions

**Fig. 4.**
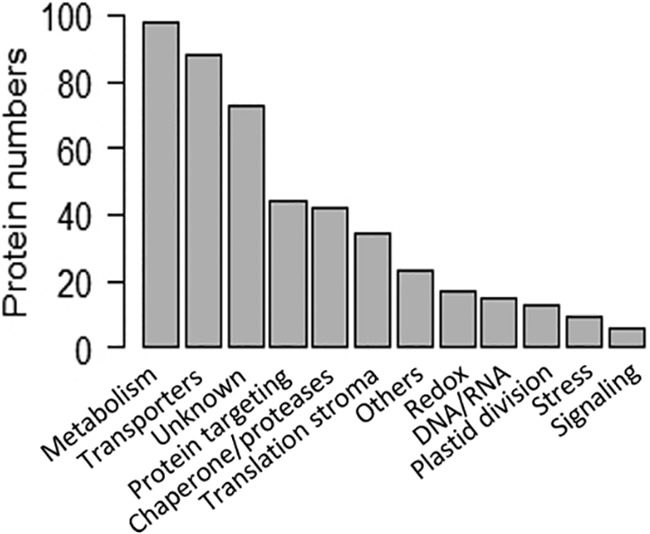
**Functional categories of the chloroplast envelope proteome as deduced from manual annotation (see supplemental Table S5, column “Curated function”).** Functional annotations of the 462 envelope proteins were collected from the appropriate literature (PubMed) and databases (Uniprot, TAIR, MapManBins…). Note that although 20% of envelope proteins were classified as “unknown,” the functions of the vast majority of proteins assigned to known functional groups were deduced from sequence similarity and remain to be demonstrated.

The in-depth manual analysis (literature, databases, predictions…) also allowed us to assign functional categories to 1269 proteins identified in purified envelope fractions, including 462 chloroplast envelope proteins (see supplemental Table S5, columns “Curated function” and “Curated description”). This allowed us to gain an overview of the functional profiles of the newly detected proteins, and to actualize functional descriptions of previously identified envelope proteins (12) using post-2010 data from the literature. As shown in Fig. 4, 100 envelope proteins were classified as metabolism actors (mostly involved in metabolic pathways linked to synthesis of lipids, hormones, vitamins and pigments) or as transporters (almost 90 members including inner and outer envelope membranes). Although the function of most envelope proteins could be deduced from sequence similarities, the functions of around 16% of envelope proteins (more than 70 proteins) remain totally unknown and unpredictable (Fig. 4). Remarkably, “chaperone and protease,” “translation stroma,” and “protein targeting” were also among the main functional categories for the envelope proteins (Fig. 4). Indeed, around 30% of total genuine envelope proteins were categorized into one of those three functional groups. Finally, many envelope proteins were classified as “redox,” “stress,” “DNA/RNA interacting proteins,” “signaling,” or “others.” Again, although some of these proteins could be classified according to similarities with known proteins or predicted functional domains, most of these proteins remain uncharacterized envelope proteins.

##### Variation of the Enrichment Factor (EF) Allows Distinguishing Genuine Envelope Proteins From Components Shared with Other Chloroplast or Cell Compartments

**Fig. 5.**
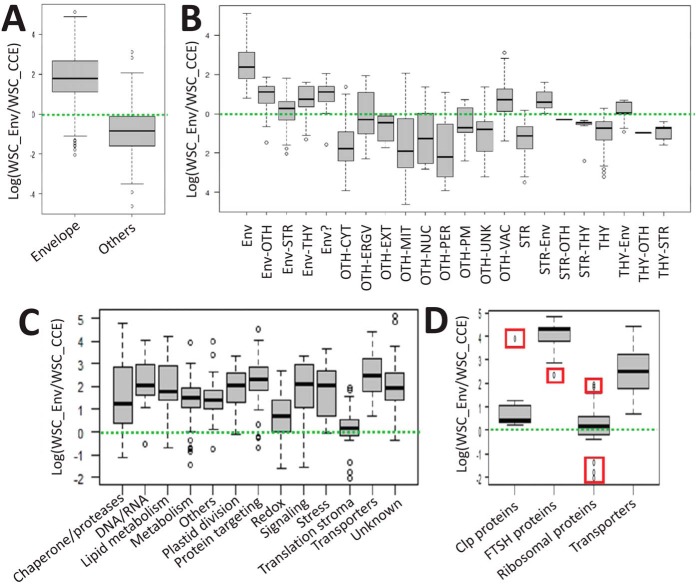
**Descriptive statistics depicting groups of EF values for cell proteins identified in purified envelope fractions (see supplemental Table S5, “this work,” columns “EFestimator” and “Simplified location (this work)”).** *A*, Box plots displaying EF variations in the chloroplast envelope proteome (average EF = 30) when compared with other proteins (average EF = 0.1). *B*, Most non-envelope cell components have lower EFs. Surprisingly, vacuolar components are slightly enriched in the purified envelope fraction with an average of 2. ENV; All envelope proteins (including outer envelope membrane components), ENV-Oth; proteins shared with other undefined plastid compartments, ENV-STR: envelope proteins shared with the stroma, ENV-Thy; envelope proteins shared with the thylakoid, ENV?; Envelope candidates; OTH-CYT; cytosol, OTH-ERGV: endoplasmic reticulum/Golgi, OTH-Ext; extracellular localization, OTH-Mit; mitochondria, OTH-Nuc; nucleus, OTH-PER; peroxisome, OTH-PM; plasma membrane, OTH-Vac; vacuole, STR; stroma, THY; thylakoid. Descriptive statistics depicting groups of EF values for functional categories at the chloroplast envelope scale. *C*, Box plots displaying EF variations across functional categories (see Fig. 4) of the chloroplast envelope proteome. *D*, Note that when assigning protein complexes (*e.g.* 14 Clp subunits 9 FtsH subunits) to a single functional category (*e.g.* here, “Chaperone and protease”), both the interquartile ranges and the degree of dispersion are reduced (see *A*). Outliers (here, ClpC2 and Ftsh7) are plotted (red squares) as individual points easily identified in supplemental Table S5 (“this work,” columns “EFestimator” and “Curated function (this work)”). Ribosomal proteins = 34 proteins, transporters = 88 proteins.

The average EF of the 462 envelope proteins is clearly above that of all non-envelope proteins detected in the purified envelope fractions (Fig. 5*A*). This was also the case when comparing envelope proteins with proteins associated with any other plastid or cell compartments (if one excludes vacuolar proteins, or stroma and thylakoid proteins shared with the envelope, see Fig. 5*B*). The average EF of genuine stroma or thylakoid proteins is far below that of stroma and thylakoid proteins shared with the envelope.

However, although assigned to a unique subcellular or subplastidial localization, the EF values were highly variable for proteins of the same group (see Fig. 5*B*). This observation could be caused by quantification artifacts resulting from proteins situated at the threshold level of MS detection (*i.e.* where very low values of spectral counts have a strong impact on EF values). This is particularly the case for proteins that were only detected in the envelope fraction. Recall that, among these proteins, the elimination of those having SWSC = 1 (supplemental Table S1) prevented the occurrence of meaningless Enrichment Factors.

We analyzed these data in more detail, not only considering envelope localization, but also functional categories (Fig. 5*C*) at the scale of the whole envelope. Again, although the median EF was in good agreement with the enrichment of these functional groups in the chloroplast envelope (“transporters” being at the top level and “translation stroma” being at the lowest level), the EF values were highly variable for proteins of the same group. This result might derive from the various subplastidial locations of the proteins from the same groups (the genuine envelope proteins having a higher EF value than the proteins shared with stroma or thylakoid sub-compartments).

We then concentrated on specific proteins known to be part of the same family or the same multi-protein complexes within one of these functional groups. As an example, starting from the 40 members (see supplemental Table S5) of the group “chaperone and proteases” (Fig. 5*C*) we calculated the median EF value (and its variation) of the 9 FtsH and 14 ClpP proteins (Fig. 5*D*). In agreement with previous observations, and according to AT_CHLORO (12), the FTSH (38) complex is exclusively localized in the envelope. This protein complex appears to be highly enriched in the envelope fraction when compared with the crude cell extract (see supplemental Table S5, column “EF estimator”). The median EF value of the FTSH complex is even stronger than that of the 88 transporters associated with the chloroplast envelope (see Fig. 5*D*). On the other hand, it was suggested in AT_CHLORO (12) that the subunits of the Clp protease complex (39, 40) are probably shared between stroma and envelope compartments (see supplemental Table S5 “This work”, columns “ENV %” and “STR %”). Interestingly, the EF values of most of these subunits are very low when compared with that of FtsH proteins. With the exception of ClpC2 (AT3G48870) which is highly enriched in the envelope fraction (EF > 50, see supplemental Table S5, column “EF estimator”, and the top outlier/red square in Fig. 5*D*), all other Clp proteins share the same intermediate EF value (average = 2) with a reduced degree of dispersion (EF between 1.3 and 3.5), *i.e.* just above a threshold level corresponding to the 34 “ribosomal proteins” from the stroma (see Fig. 5*D*). To cite a different example, within the functional category “lipid metabolism,” two members of the Acetyl-CoA carboxylase complex (ACCDa, AT2G38040 and ACCDb, AtCg00500) were mostly detected within envelope membranes, whereas two others (BCCP1, At5g16390 and BCCP2, At5g15530) were previously shown to be shared between envelope and stroma (12). In good agreement with these previous observations, the EF values of ACCDa and ACCDb are higher (24.3 and 13.6, respectively), when compared with the ones of BCCP1 and BCCP2 (3.5 and 0, respectively. BCCP2 was not even detected in the present work, see supplemental Table S5, lanes 73 to 75). Again, in the functional category “lipid metabolism,” the six members of the pyruvate dehydrogenase multi-enzyme complex, were previously shown (12) to be shared by envelope and stroma (see supplemental Table S5, lanes 97 to 102). Out of the six subunits of this complex, five share the same intermediate EF (average of 4), and only one member (ptLPD1, At3g16950), which was only detected in purified envelope fractions, has a higher EF of 24 (see supplemental Table S5, lane 118).

To summarize, even at the envelope scale, a good correlation is observed between high EF values and unique envelope localization. Likewise, intermediate EF values are correlated with previously observed sharing of a protein between the envelope and other plastid compartments.

##### Defining EF Threshold Levels to Exclude Putative Plastid and Cell Contaminants

**Fig. 6.**
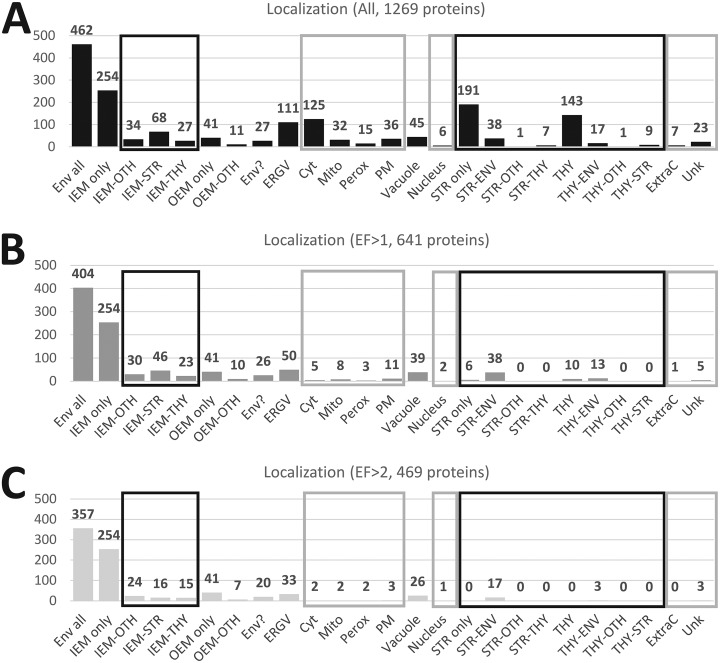
**Increasing the EF threshold level to 1 or even 2 reduces the number of envelope proteins shared with other compartments and excludes most other plastid and cell components (see supplemental Table S5, *A* and *B*, “Only EF>1” or *C*, “Only EF>2”).** Note that most (84% for EF>1 or 95% for EF>2) of the 406 plastid proteins (right black squares) localized in the stroma and thylakoid are excluded using a threshold of 1 or 2 when compared with whole data. This is also true for most (85% for EF>1 or 95% for EF>2) of the 244 proteins (gray squares) from the cytosol, mitochondria, peroxisome, plasma membrane, nucleus, extracellular space, and unknown compartment. However, note that even an EF threshold of 2 does not remove non-negligible parts of ER/Golgi (30%) and vacuolar (58%) components.

From the above-cited observation, the EF value appears reliable. It was tempting to define a tentative threshold level for EF that would allow discrimination of genuine envelope proteins from contaminants derived from other plastid or cell compartments. As discussed above, most envelope proteins are characterized by a high enrichment factor with an average value of 13 (Fig. 3B). Most other plastid or cell components have an average EF value of 1, except for vacuolar proteins. These proteins were revealed to be slightly enriched in the envelope fraction, having a higher EF value than other cell components (average of 3.3 higher; Fig. 3*B*). Remarkably, when increasing the EF threshold to 1 or 2 (see supplemental Table S5, “Only EF>1” and “Only EF>2”), most (85% for EF>1 or 95% for EF>2) of the 244 proteins from the cytosol, mitochondria, peroxisome, plasma membrane, nucleus, extracellular and unknown proteins are excluded (see Fig. 6*B* and 6*C*, light gray square). This is also true for most (84% for EF>1 or 95% for EF>2) of the 406 plastid proteins, localized in stroma and thylakoid, which are excluded using a threshold of 1 or 2 (see Fig. 6*B* and 6*C*, right black square). However, these tentative threshold levels, which eliminate most envelope contaminants, reduces the number of the 129 envelope proteins (33% for EF>1 or 57% for EF>2) that are shared with other plastid compartments (see Fig. 6*B* and 6*C*, left black square). This observation thus raises the question of the localization of these proteins shared by envelope and other plastid compartments, and of the relevance of these intermediate EF values.

##### Intermediate EF Values Are Compatible with Genuine Envelope Localization

**Fig. 7.**
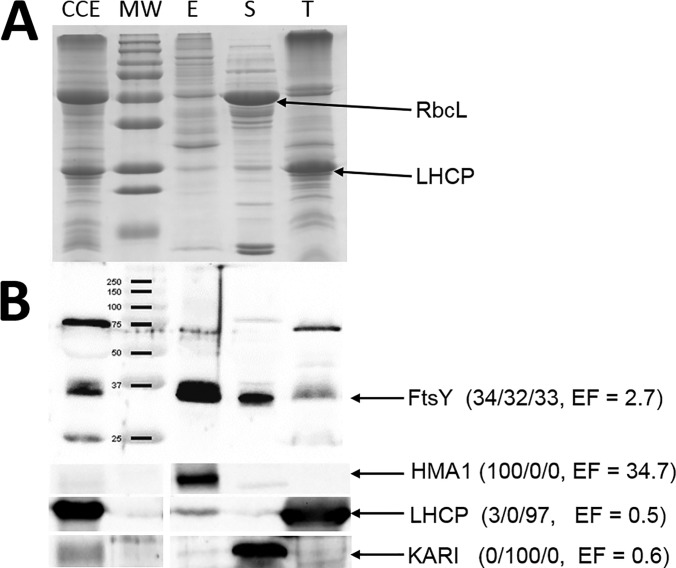
**Intermediate EF values are compatible with genuine envelope localization.** *A*, Representative SDS-PAGE analysis. Each lane contains 15 μg of proteins. CCE, crude cell extract; MW, molecular weight; E, envelope fraction, S, stroma; T, thylakoid. RbcL, Large Subunit of Rubisco. LHCP, Light harvesting complex proteins. *B*, Validation of the multiple subplastidial localizations of FtsY protein by Western blotting. Experiment performed on Arabidopsis plants, using FtsY antibody. The expected MW of the mature form (*i.e.* after cleavage of its plastid transit peptide) of FtsY is 35.1 kDa. Values between brackets are % abundance in the envelope, stroma, and thylakoids, according to Ferro *et al.* (12), and the EF value (this work). As expected from previous data (see AT_CHLORO database), FtsY is shared between the three chloroplast sub-compartments (34% in Envelope, 32% in Stroma and 33% in Thylakoid). Note that although unambiguously present in the envelope membrane, the EF value (2.7) of FtsY is far below the average EF value of envelope proteins (*i.e.* 13, see Fig. 3). LHCP, Light harvesting complex proteins. HMA1, Heavy Metal ATPase 1. KARI, Ketol-acid reducto-isomerase.

To answer this question, we chose to analyze the subcellular and subplastidial localization of several proteins. The protein FtsY (AT2G45770) (41), was previously shown by Ferro et *al.* (12) to be distributed between the three chloroplast sub-compartments (34% in envelope, 32% in stroma and 33% in thylakoid) (see supplemental Table S5, lane 146). In agreement with these previous observations, this protein has an intermediate EF value of 2.7; quite compatible with its shared localization. A Western blotting was performed on Arabidopsis crude cell extract and all three purified plastid sub-compartments using a polyclonal antibody raised against FtsY (Fig. 7). As expected, FtsY was detected in all three chloroplast compartments, *i.e.* envelope, stroma and thylakoid, and its signal in the purified envelope was enriched when compared with the crude cell extract. As internal controls, the envelope marker, heavy metal ATPase (HMA1; At4t37270) (20), was exclusively localized in purified envelope fractions (EF = 34.7). The ketol-acid reducto-isomerase KARI (At3t58610, EF = 0.6) was associated with the stroma (12). The thylakoid markers, light harvesting complex family of proteins (LHCP) (average EF of LHCPs = 0.5), were only associated with the thylakoid (Fig. 7). Altogether, these observations confirm the correlation between each respective EF value and the subplastidial localizations of these proteins. In other words, although the EF value (2.7) of FtsY is far below the average EF value of envelope proteins (*i.e.* above 13, see Fig. 3B), this protein is unambiguously a component of the envelope membrane.

**Fig. 8.**
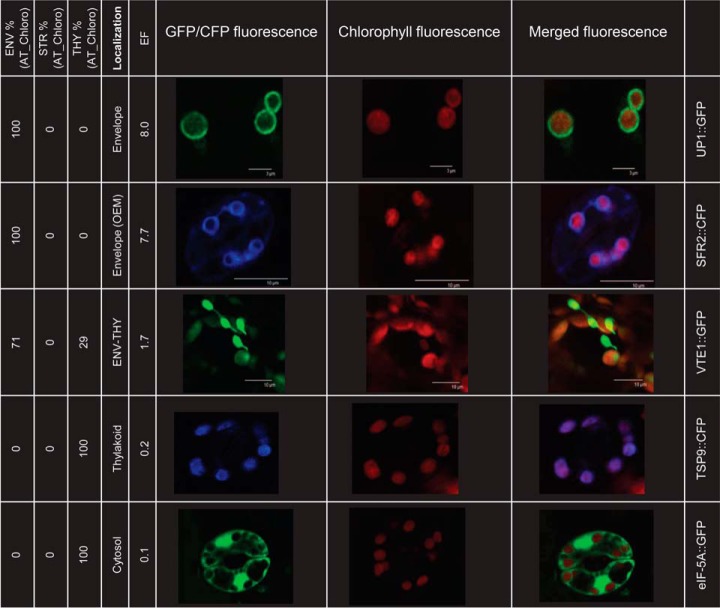
**Illustration of the correlation between enrichment factor (EF) and subcellular or subplastidial localizations of proteins deduced from *in planta* expression of GFP/CFP fusions.** To investigate protein localizations, Arabidopsis plants stably expressing UP1-GFP (envelope only, intermediate EF 8.0), SFR2-CFP (outer envelope membrane, intermediate EF 7.7), VTE1-GFP (shared between envelope and thylakoid, intermediate EF 1.7), TSP9-CFP (thylakoid only, low EF 0.2), and eIF-5A-GFP (cytosol, low EF 0.2) were constructed. From left to right: data from AT_CHLORO (% in envelope, stroma, and thylakoid), deduced subcellular and subplastidial localization, EF deduced from this work, fluorescence of GFP or CFP fusion proteins, chlorophyll auto-fluorescence, merge of GFP/CFP and chlorophyll fluorescences, and protein names. Note that the fluorescence of SFR2-CFP reveals that SFR2, which previous data have shown is localized in the outer envelope membrane (43), might also be shared with non-plastid structures, thus explaining its intermediate EF value. One would expect a higher EF value for UP1, which is localized to the envelope only. However, note that UP1 was not detected in the crude cell extract sample and its EF might have been underestimated. The perfect correlation between TSP9-CFP fluorescence and chlorophyll autofluorescence agrees with the thylakoid localization of the protein and supports its lower EF value of 0.2. Note that although correlations between EF values and subcellular localizations were obtained, GFP fusion overexpression systems might not accurately reflect the localizations of the endogenous proteins.

As an alternative to immunodetection of proteins, we also examined the subcellular and subplastidial localization of several other proteins *in planta*. WT Arabidopsis plants stably expressing UP1::GFP (At1g11320, EF = 8.0) (42), SFR2::GFP (At3g06510, EF = 7.7) (43), VTE1::GFP (At4g32770, EF = 1.7) (44), TSP9::CFP (At3g47070, EF = 0.2) (45) and eIF-5A::GFP (At1g26630, EF = 0.1) (46, 47) were constructed to assess the compatibility between localization of the proteins and their EF values. As expected, the negative control, eIF-5A, which has a very low EF value (0.1), was distributed in the cytosol. In agreement with its very low EF value of 0.2, the thylakoid soluble phosphoprotein, TSP9 (45), was distributed in the thylakoid subplastidial compartment, as evidenced by its perfect co-localization with chlorophyll auto-fluorescence (Fig. 8). Likewise, the protein encoded by At1g11320, annotated UP1 (40) was unambiguously localized in the chloroplast envelope (12) (Fig. 8). Therefore, these results agree with the low and high EF values of TSP9 and UP1, respectively (Fig. 8). VTE1 (42), the tocopherol cyclase involved in tocopherol synthesis, was suggested to be dually localized in the chloroplast envelope and thylakoid (12). The EF value of VTE1 is 1.7, thus 8 times higher than TSP9 (EF = 0.2), which is only localized in the thylakoid, but far below the EF of UP1 (EF = 8.0). Analyzing the subcellular localization of VTE1::GFP in Arabidopsis plant tissues (Fig. 8) revealed the existence of long extensions into the cytosol called stromules (48). According to Breuers *et al.* (48), these stromules are membrane proliferations derived from the envelope membrane, suggesting that VTE1 is potentially localized in this membrane system. However, when compared with UP1 images, VTE1 fluorescence also correlates with chlorophyll auto-fluorescence, suggesting that VTE1 is also localized in the thylakoid membranes. Finally, we also analyzed the localization of SFR2 (Fig. 8), an outer envelope glycosyl hydrolase with a demonstrated role in protecting chloroplasts against freezing-induced damage in Arabidopsis (43). Interestingly, in agreement with its expected envelope localization, images obtained for SFR2 are like that of UP1 (Fig. 8). Indeed, plastids were surrounded by SFR2::CFP fluorescence. There was little, if any, correlation between chlorophyll autofluorescence and the fluorescence of SFR2::CFP. Accordingly, the high EF value (7.7) of SFR2 compared with VTE1 (1.7) suggests that SFR2 might not be shared between the plastid envelope and other plastid sub-compartments.

##### High EF Threshold Levels Exclude Most Envelope Proteins Shared with Other Compartments, but Does Not Remove Some ER/Golgi and Vacuolar Components

As discussed above, increasing the EF threshold level excludes most other plastid and cell contaminants. However, we noticed that although high EF threshold levels (*i.e.* EF > 2) exclude most proteins from the stroma, thylakoid, cytosol, mitochondria, peroxisome, plasma membrane, nucleus, and extracellular proteins, they do not exclude non-negligible parts of ER/Golgi (30%) and vacuolar (58%) components (see Fig. 6 and supplemental Table S5, “Only EF>1”, “Only EF>2”). Further, when calculating the impact of higher EF thresholds on the average EF values of the remaining proteins, we noticed high average EF values of the 33 remaining ER/Golgi proteins and 26 remaining vacuolar proteins (see supplemental Fig. S5, black squares). These average EF values are, respectively, 4 and 3 for the remaining ER/Golgi and vacuolar proteins; *i.e.* above genuine chloroplast envelope proteins (like FTSY, see above) that are shared with other compartments (see Fig. 7).

**Fig. 9.**
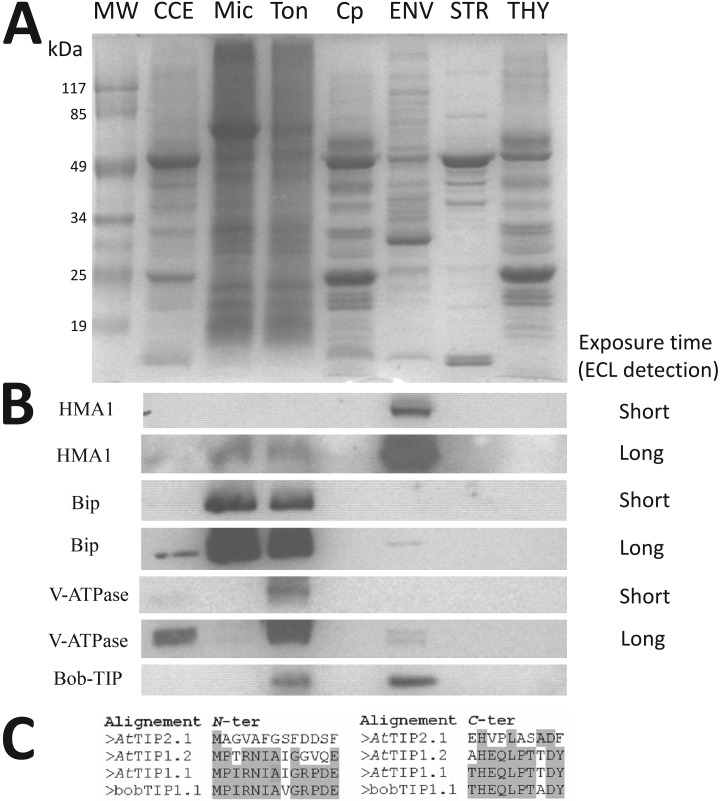
**Detection of vacuolar and ER/Golgi components in purified envelope fractions is partially explained by cross-contamination.** *A*, SDS-PAGE analysis. Each lane contains 15 μg of proteins. MW, molecular weight; CCE, crude leaf extract; Mic, microsomal fraction, Ton, tonoplast fraction, Cp, Percoll-purified chloroplast, E, envelope fraction, S, stroma; T, thylakoid. *B*, Analysis of envelope contamination with markers from ER/Golgi and tonoplast components. Validation of the enrichment of HMA1 (envelope marker) when compared with CCE. As expected, Bip (ER marker) is enriched in microsomal fraction and contaminates purified tonoplast fractions. The same is true for V-ATPAse (tonoplast marker), which is strongly enriched in the tonoplast fraction. Note that both Bip and V-ATPAse signals were barely detectable in purified envelope fractions. However, these signals are not enriched when compared with CCE. Surprisingly, the signal detected using the bob-TIP antibody (raised against *N*- and *C*-ter of the cauliflower TIP1.1, a tonoplast marker), is similar in the tonoplast and purified envelope fractions, thus suggesting specific cross-contamination or dual localization. Note that this protein was the only TIP isoform that was previously detected in envelope fractions by both Simm *et al.* (14) and Ferro *et al.* (12). *C*, Alignment of *N*- and *C*-ter of cauliflower bobTIP1.1, supporting detection of the TIP1.1 protein in Arabidopsis samples.

Thus, we questioned whether the presence of some vacuolar and ER/Golgi components in purified envelope fractions could be explained by specific cross-contaminations. As shown in Fig. 9*A*, we purified crude cell extracts from leaf, microsomes, tonoplast, Percoll-purified chloroplasts, envelope, stroma, and thylakoids. These fractions were then tested for cross-contamination using markers for the envelope, ER/Golgi and tonoplast compartments (Fig. 9*B*). As expected, HMA1 (envelope marker) was strongly enriched when compared with the crude cell extract. Only long exposure times (Long ECL detection, see Fig. 9*B*) allowed the detection of its signal within microsomes and tonoplast lanes. The same was true for Bip (ER marker), which was strongly enriched in the microsomal fraction when compared with the crude cell extract. It also contaminates purified tonoplast fractions. Similarly, V-ATPAse (tonoplast marker) is strongly enriched in the purified tonoplast fraction when compared with the crude cell extract. Bip and V-ATPAse signals were undetectable in purified envelope fractions, and only long exposure times (Long ECL detection, see Fig. 9*B*) allowed the detection of their signals within the envelope lane. At this detection level, we could notice that both Bip and V-ATPAse signals were not enriched in the envelope when compared with the crude cell extract (Fig. 9*B*). In other words, even if one expects to detect minute amounts of ER/Golgi and vacuolar proteins in purified envelope fractions, these proteins should not be as enriched as the proteins that are specifically localized to the envelope membrane. However, from these observations, we cannot rule out the possibility that some abundant markers of ER, Golgi, or vacuole could get higher EF values than envelope proteins that are shared with other plastid or cell compartments. Only specific assays such as immuno-localization and *in planta* analyses can reveal more detailed information about the sharing of ER, Golgi, or vacuole proteins with the chloroplast envelope, even if they are repeatedly detected in one of these compartments.

Surprisingly, during the course of these analyses, the signal detected using the bob-TIP antibody (raised against *N*- and *C*-ter of the cauliflower tonoplast intrinsic protein TIP1.1, another tonoplast marker, see Fig. 9*C*) was enriched in the tonoplast relative to the crude cell extract, but was also detected at a similar level in purified envelope fractions (Fig. 9*B*). It is important to note here that TIP1.1 (At2g36830) was the only TIP isoform previously detected in envelope fraction by both Simm *et al.* (14) and Ferro *et al.* (12). It was repeatedly localized in the tonoplast using *in planta* expression of GFP fusions, but was rarely detected in purified tonoplast fractions (see supplemental Table S5, lane 793, columns “Location GFP” and “Experimental evidence”). Altogether, these observations suggest that a specific cross-contamination or a dual localization of this expected tonoplast marker in the chloroplast envelope cannot be excluded.

## DISCUSSION

The aim of the present study was to revisit the chloroplast envelope proteome, taking advantage of improving subcellular proteome databases and MS sensitivity, to perform a quantitative proteome study comparing purified envelope fractions and crude cell extracts. As expected, many more proteins were detected, compared with previous studies, including well-known but previously undetected chloroplast envelope proteins (see Table I and supplemental Table S5 “This work”). Additionally, we detected many proteins deriving from other cell compartments (*i.e.* putative contaminants, see Fig. 3) with a specific overrepresentation of proteins from the cytosol, the ER/Golgi, and the vacuole when compared with previous studies (see Fig. 3 and supplemental Fig. S4, and Fig. 6). Accessibility to a quantitative approach (EF parameter), thanks to improved MS sensitivity, allowed us to revisit the chloroplast envelope proteome and easily exclude most contaminants that were derived from other plastid or cell compartments (see Fig. 3 and Fig. 5 to 6). Thus, the overall quality of our present proteome was not diminished by the detection of proteins that are highly abundant in other cell compartments.

### 

#### 

##### Improved Annotation of Proteins

Aside from the identification of novel envelope components (supplemental Fig. S4) we also provide a detailed manual annotation based on recent characterization of some of these proteins (see supplemental Table S5, column “curated description”). These recent annotations (*i.e.* up to early 2018) strongly revisited the information present in AT_CHLORO database, which inventories the list of all MS-identified envelope proteins (12). Indeed, 185 of the 413 envelope proteins (ENV), 22 of the 54 outer envelope membrane proteins (OEM), and 42 of the 46-envelope candidate (ENV?) categories could be redefined thanks to recent data. The data includes their functional characterization (or of a member of their family), similarities with characterized orthologous proteins from other species, or even identification of functional domains predicting specific functions. To cite a few examples: a chloroquine-resistance transporter-like protein (At5g12170, Env_only) was shown to be required for glutathione homeostasis and stress responses (49). BASS2 (At2g26900, former called IEP36) is now characterized as a plastidial sodium-dependent pyruvate transporter (50). Members of the FAX family (At3g57280, At2g38550, formerly called HP26-like proteins) were recently characterized as novel membrane proteins mediating plastid fatty acid export (51). Even if their definitive role in targeting proteins to plastids is still a matter of debate (52, 53), Tic214 (AtCg01130, formerly called Ycf1) and Tic56 (At5g01590, formerly called HP65) were recently shown to participate in a 1-MDa protein complex comprising Tic214, Tic56, Tic100 (At5g22640) and Tic20-I (At1g04940), at the inner chloroplast envelope membrane (54, 55). TIC236 (At2g25660), an inner envelope membrane protein was recently shown to bind the outer envelope membrane channel TOC75 thus linking the TOC and TIC translocons in a TOC-TIC supercomplex (56). This protein, detected in the present work (see Table I), was not identified by earlier proteomic studies (12, 14). VTE6 (At1g78620, formerly called HP34) was demonstrated to be a phytyl-phosphate kinase catalyzing the conversion of phytyl-monophosphate to phytyl-diphosphate (57). ceQORH (At4g13010), a former putative quinone oxidoreductase, was recently demonstrated to reduce long-chain, stress-related, oxidized lipids (58) and its structure was later solved (59).

As previously observed, SecA2 (At1g21650) and SCY2 (At2g31530) were only detected in the envelope fraction and have relatively high EF values (28 and 8, respectively). Although SecA2 was previously detected in the envelope fraction using a proteomics approach (12), SCY2 was identified for the first time in the present work. Both proteins were recently described as components of a Sec translocon, localized in the inner envelope membrane, which likely integrates a subset of inner envelope membrane proteins into the envelope (60–62).

Altogether, these data limited the number of envelope components that were missing in previous analyses that were targeted at envelope fractions. However, although many new or expected but previously undetected envelope proteins were identified during this work (see supplemental Table S5, column “curated description”), expected envelope components are still lacking. For example, this is the case of numerous envelope transporters that are expected to control exchanges of ions and metabolites across the chloroplast envelope (for a review, see (9)), but whose molecular identities remains to be determined.

We also detected several chloroplast-encoded proteins that are enriched in envelope fractions: the protease ClpP1 (ATCG00670), the beta chain of the acetyl-CoA carboxylase ACCDb (ATCG00500), Ycf1 (ATCG01130), and Ycf2 (ATCG00860, only detected in envelope with high EF) proteins. However, the chloroplast-encoded protein, Ycf10 or CemA (AtCg00530) (63), which, to our knowledge has never been detected using a MS-based approach targeting envelope or any other chloroplast sub-compartment was, again, not detected here. To cite a few more, some expected components of the TIC (*i.e.* Tic20-IV, At4g03320) and Toc (Toc75-iV, At4g09080) complexes were not detected during this work.

The present analysis also raises numerous questions about the genuine subcellular localizations of some identified proteins. Because of space constraints, it was not possible to describe all peculiar cases that were classified during manual annotation of the 1269 proteins detected in purified envelope fractions. Below, we will discuss a few interesting cases that might deserve specific attention.

##### A Few Examples of Genuine Envelope Proteins Whose EF Values Help Predict Subplastidial Localization

Unexpectedly, we noticed that some genuine plastid and expected envelope proteins were not enriched in the envelope fraction (Fig. 3*B*). Most of these proteins are most probably shared between envelope and other subcellular or subplastidial compartment. For example, four allene oxide cyclase (AOC1, AOC2, AOC3 and AOC4) were previously described as enzymes of the jasmonate pathway that are required for the jasmonate biosynthesis (64, 65). Together with AOS (allene oxide synthase), four AOC isoforms were previously demonstrated to be targeted to plastids (65) and three AOC1, 2 and 3 isoforms were previously detected in purified envelope fractions (12). AOC4, which was not previously identified by MS-based approaches in the chloroplast envelope fraction, was also detected during this work. In our experiment, AOC1, AOC2 and AOC4 were detected with an EF below to 1. AOC3 is the only isoform of AOC that was detected only in the envelope (intermediate EF value of 3.3) (see supplemental Table S5, lanes 65–68). This observation suggests that at least all three other isoforms might be shared with other plastid compartments.

Transporters were also deeply analyzed here. Interestingly, as cited above, several members of this functional category were given a substrate recently. The present work also helped determine their subplastidial locations. As controls, KEA3 (At4g04850), a recently described thylakoid K(+) efflux antiporter (66) has an expected low EF value of 0.3 (see supplemental Table S5, lane 1220). On the other hand, its close homologues, KEA1 (At1g01790) and KEA2 (At4g00630), known envelope transporters (67, 68), have EF values of 8 and 15 respectively (see supplemental Table S5, lanes 286–287). We previously provided data (12) that contradicted previous information that described TAAC (At5g01500) as a thylakoid ATP/ADP carrier. Indeed, this protein was previously detected in purified envelope fraction, but absent from purified thylakoid membranes (12). In the present work, as before, the protein was only detected in envelope fractions (see supplemental Table S5, lanes 292) with a high EF value of 37.1; definitively ruling out its association with thylakoid membranes. The same is true for the thylakoid transporter ANTR1 (At2g29650). Again, this protein was described as a thylakoid Na^+^-dependent phosphate transporter from Arabidopsis (see (12)) whereas our previous data (proteomics and Western blotting analyses using specific polyclonal antibodies, see (12)) clearly demonstrated that this protein was associated with the envelope. Again, the present work strongly supports our previous data, because this protein was only detected in envelope fractions (see supplemental Table S5, lanes 312) with an EF value of 8.9; thus also ruling out its association with thylakoid membranes. The function of this protein was recently revisited, and it was demonstrated to catalyze ascorbate transport (69). This ascorbate transporter is thus, without a doubt, associated with the chloroplast envelope.

The correlation between EF values of proteins that are involved in the same multisubunit complexes was discussed above for Clp and FtsH subunits (Fig. 5*D*). Evidence that multiple different Toc complexes are present in plastids, and hypotheses concerning the client specificities of these Toc complexes, can be traced back to the early 1990s (70, 71). In a recent model proposed by Nakai (72), Toc34 (At5g05000), Toc132 (At2g16640), and Toc120 (At3g16620) are components of a first TOC complex required for import of “non-photosynthetic” proteins, whereas Toc33 (At1g02280) and Toc159 (At4g02510) are part of a second “photosynthetic” TOC complex devoted to import of proteins linked to photosynthesis. Interestingly, Toc33 (EF = 16.5) and Toc159 (EF = 11.5) that are part of the same “photosynthetic-type” TOC complex have similar intermediate EF values, whereas the three components of the “non-photosynthetic-type” TOC complex, Toc34 (EF = 22.7), Toc132 (EF = 38.4) and Toc120 (EF = 5.2), showed less homogenous EFs values, most likely because the latter two were only detected in the envelope sample. In the same model proposed by Nakai (72), the “photosynthetic type” major TIC complex would be made of Tic56 (At5g01590), Tic100 (At5g22640), Tic20-I (At1g04940), and Tic214/Ycf1 (AtCg01130). Interestingly, Tic56 (EF = 56.1), Tic100 (Env_only, EF = 90.2), Tic20–1 (Env_only, EF = 10.9), and Tic 214/Ycf1 (EF = 41.9) have higher EF values than all other detected Tic subunits (except Tic55-IV, see supplemental Table S5, lanes 150–163).

It is important, however, to note that the crude cell extract used during this study contains plastid proteins derived from all leaf tissues (including epidermal cells), whereas purified envelope fractions mostly derive from photosynthetic chloroplasts extracted from parenchymal cell. Although probably limited, this small bias might impact the EF values of some genuine envelope proteins that are part of the same complex. However, if one excludes the higher EFs of Toc34 (22.7) and Toc132 (38.4), most other Toc subunit share similar EF values with a relatively low degree of dispersion (around 14) (EF Toc33 = 16.5, EF TOC64-III = 14.6, EF Toc75-III = 9.6, EF Toc75-V = 16.9, EF Toc90 (9.0), EF Toc120 = 5.2, EF Toc159 = 11.5).

##### Cross-contamination by Mitochondria Components

Qualitative proteome analyses identified 32 mitochondrial proteins (Fig. 3) with an expected low EF (average EF = 0.7). With very few exceptions (At3g27930 and At1g28690), these proteins were also repeatedly detected in purified mitochondria during previous MS-based studies (see supplemental Table S5, lanes 700–731). The protein with the highest EF value (EF = 7.9, only detected in purified envelope fractions) was an ethanolamine-phosphate cytidylyltransferase called PECT1 (At2g38670), a rate-limiting enzyme in phosphatidylethanolamine biosynthesis. Using enzymatic measurement, MS-based analyses, GFP fusions, and *in planta* analyses (see (73) and references therein), this protein was previously associated to the mitochondrial periphery, most likely at the interface between the cytosol and the outer membrane of the mitochondrion. Interestingly, this protein was also detected in purified envelope membranes from *Medicago sativa* (14), thus supporting our data and suggesting that PECT1 might also interact with the plastid surface. However, we chose to maintain this protein in the list of mitochondrial components. The same was true for a NADH-ubiquinone oxidoreductase (At4g16450) with the subsequent highest EF value (EF = 3.9) for a mitochondrial protein. Interestingly, PECT1 and this NADPH-ubiquinone oxidoreductase were both detected in purified envelope membranes during a recent study by Simm *et al.* (14).

All other known mitochondrial proteins, known to be shared by mitochondria and chloroplasts (dual targeted), appear as envelope components in the supplemental Table S5. We even chose to include a few previously known mitochondria proteins in the list of envelope components that were shown to be highly enriched in purified envelope fractions. For example, MSL1 (At4g00290, also called MscC) was recently shown to be a mechanosensitive ion channel that dissipates mitochondrial membrane potential and maintains redox homeostasis in mitochondria during abiotic stress (75). During this work, this protein was only detected in the chloroplast envelope (see supplemental Table S5, lane 277) with a very strong EF of 27.8, *i.e.* far above the 0.7 average impact factor of mitochondrial proteins. The same is true for ATM3 (At5g58270, also called ABCB25, STA1 or STARIK1). This protein was recently proposed to export glutathione polysulfide, containing glutathione and persulfide, from mitochondria, for iron-sulfur cluster assembly in the cytosol (75). Again, we detected this protein in the chloroplast envelope (see supplemental Table S5, lane 266) with an EF of 66.4, far above the average impact factor of genuine envelope proteins.

The above-cited examples raise the question of the genuine localizations of these proteins or at least, potentially suggest sharing of these mitochondrial proteins with the chloroplast envelope. In other words, the application of the present analyses opens up interesting possibilities to answer targeted questions about the subcellular localizations of these proteins.

##### Cross-contamination by Vacuolar Components

Qualitative proteome analyses identified various vacuolar and particularly, tonoplast proteins with surprisingly high EF values (see supplemental Fig. S5). This can be explained by specific co-purification of the two light membrane systems (envelope and tonoplast). Alternatively, this could result from the sharing of specific tonoplast proteins with envelope membranes. The TIP1.1 (At2g36830, see supplemental Table S5, lane 793) might be an example of this, even if its EF (2.0) is relatively low. Similarly, autoinhibited Ca^2+^-ATPases ACA4 (At2g41560) and ACA11 (At3g57330, see supplemental Table S5, lanes 797–798) were highly enriched or only detected in envelope fractions during this work (respective EF values of 17 and 7.7). Although repeatedly detected in purified tonoplast fractions, ACA11 is the only tonoplast protein containing a predicted (TargetP) chloroplast transit peptide. The vacuolar H+-ATPase (V-ATPase) is a membrane-bound multisubunit enzyme complex composed of at least 14 different subunits. Note that the subunit, VATB3 (At1g20260, see supplemental Table S5, lane 805), has only been detected in the envelope fraction and has the highest EF value among tonoplast proteins (EF = 22.3, see highest outlier of the “Vacuole” group in Fig. 5*B*). In fact, the three B subunits (AtVAB1, AtVAB2, and AtVAB3) of Arabidopsis V-ATPase were recently shown to play distinct roles in Arabidopsis cells besides their known roles as vacuolar ATPAse subunits (76). Indeed, AtVABs subunits were demonstrated to bind to and co-localize with F-actin to form higher order structures and stabilize actin filaments *in vitro*. In addition, the AtVABs also show different degrees of activity in capping the barbed ends of actin filaments and these activities were not regulated by calcium. AtVAB1 and AtVAB3 inhibited depolymerization significantly, whereas AtVAB2 weakly inhibited actin depolymerization. In plant cells, the formation of higher-order actin structures, such as bundles and cables, is crucial to stabilize the organization of actin strands and maintain the overall cellular architecture. A subset of overlapping activities of AtVABs may be involved in stabilizing long actin filaments. Interestingly, in plant cells, plastids are closely associated with actin microfilaments. A direct interaction of plastids with the actin cytoskeleton has been postulated to anchor chloroplasts at appropriate intracellular positions, to support chloroplast light-intensity dependent movement, to facilitate plastid stromule mobility and to participate in gravity perception. The known proteins implicated in plastid-actin interaction are CHUP1, an outer envelope membrane protein that is essential for chloroplast anchorage to the plasma membrane, myosin XI proteins that play a role in stromule movement and in gravitropism, and Toc159, a component of the TOC complex (for a review, see (77)). Interestingly, actin (At5g09810), myosin (At1G64330), or tubulin subunits (At5g19770 and At5g62690), CHUP1 (At3g25690) and Toc159 (At4g02510), were also detected during this work (see supplemental Table S5, lanes 661–664, and lanes 437 and 451, respectively). Although actin and tubulin subunits share the same low EF of 0.1, values for myosin, CHUP1 and Toc159 are intermediate to high (2.0, 3.6 and 11.5, respectively). These values are below the average EF of envelope proteins (*i.e.* 13), but far above the average EF of other cytosol components (*i.e.* 0.3), and thus, in agreement with a putative shared localization between envelope and other cell compartments.

##### Cross-contamination by ER/Golgi Components

As shown in Fig. 3 and supplemental Fig. S4, many of the newly detected proteins in the purified chloroplast envelope fraction were assigned to the endoplasmic reticulum (ER) and the Golgi. Although their presence in purified envelope fractions might result from isodensity of light membrane vesicles (in the original sucrose gradient) and thus cross contaminate the envelope fraction, recent data suggest that some of these proteins potentially interact with the outer chloroplast envelope and, thus, are not true contaminants. Indeed, a few chloroplast proteins were shown to transit through the ER *en route* to the chloroplast (78). The same is true for lipid vesicular trafficking, which was proposed to transit through membrane contact sites that connect both organelles (for a review, see (79)). Thus, some of the proteins detected during this work could be required for such processes. Glycerolipid synthesis in plant cells is characterized by an important trafficking of lipids between the ER and chloroplasts. A list of the main known or putative Arabidopsis proteins playing a role in lipid transfer between plastid and ER was recently established (79). This short list is composed of proteins from the chloroplast IEM or OEM, the cytosol, and the ER. During this work, we detected members from all involved cell compartments: *i*) the Phospholipase D PLDα1 (At3g15730, EF = 0.04) from the cytosol, *ii*) the two long chain acyl-CoA synthetases LACS4 (At4g23850, EF = 0.8) and LACS8 (At2g04350, EF = 0.1) from the ER, *iii*) the fatty acid translocator FAX1 (At3g57280, EF = 4.3), lipid translocators TGD1 (At1g19800, Env_only, EF = 21.8), TGD2 (At3g20320, EF = 7.8) and TGD3 (At1g65410, EF = 28.5), the MGDG synthase, MGD1 (At4g31780, Env_only, EF = 37.8), and the three lipid desaturases that were only detected in the envelope, FAD6 (At4g30950, EF = 68.3), FAD7 (At3g11170, EF = 43.2) and FAD8 (At5g05580, EF = 5.3) from the chloroplast IEM (but not FAD2 and FAD3 from the ER), and the lipid translocator LPTD1 or TGD4 (At3g06960, EF = 6.7) from the chloroplast OEM (whose subcellular localization was unclear, chloroplast-associated or ER in (78)) but which was classified as an OEM protein during this work thanks to its EF value of 6.7, which agrees with recently published data (80). In other words, the correlation between EF values of the known or predicted locations of the main actors in the ER-to-chloroplast lipid trafficking process was robust.

##### Cross-contamination by Cytosol Components

All cytosolic proteins identified during this work showed a very low EF (mean = 0.3; Fig. 3). One exception could be the ALBA protein which was only detected in the envelope with an intermediate EF value (At1g76010, EF = 3.96). This protein was previously identified during the determination of the mRNA-binding proteome of Arabidopsis etiolated seedlings (81). However, because this protein was previously detected several times in the cytosol, or as a cytoskeleton binding protein using MS-based approaches, it was maintained in the list of cytosol components. Interestingly, 49 members of the cytosol translation machinery were also detected in purified envelope fractions during this work. This includes several initiation and elongation factors, but also 42 subunits of the ribosome. None of these proteins were enriched in purified envelope membranes. However, the presence of cytosolic ribosomes on the surface of mitochondria was recently demonstrated, highlighting how protein synthesis in the cytosol may be coupled with protein targeting to the organelle and strongly supporting a potential role for cotranslational import (82). Presence of this mechanism at the chloroplast surface would explain the detection of these ribosomal proteins and their sharing with other cell compartments. We also detected HPL1 (At4g15440), a hydroperoxide lyase that, whereas cytosolic, was previously proposed to be involved in the degradation of lipid catabolites produced in the chloroplast envelope (83, 84). In agreement with the previous work of Simm *et al.* (14), we also detected this protein in purified envelope fractions (see supplemental Table S5, lane 425). The low EF value of HPL1 (1.1) might result from a transient interaction of this protein with the outer surface of the chloroplast, implying that the protein is shared by the cytosol and the outer chloroplast envelope membrane. It is worth mentioning here that important envelope-linked functions of abundant cytosolic proteins might be overlooked as a result of their low EF value. For example, the CDC48 protein (At3g09840) was never detected during earlier proteomic studies targeting the chloroplast envelope (see supplemental Table S5, lane 542). Further, the very low EF value of CDC48 (0.1) would predict that this cytosolic protein contaminates our envelope preparations. However, this protein was recently described to act at the envelope in the same pathway as the ubiquitin E3 ligase SP1, which regulates Toc translocase components (85). In other words, although excluded from the list of envelope components, this protein might be shared by the cytosol and the outer chloroplast envelope membrane, and its low EF value might result from a transient interaction of this protein with the outer surface of the chloroplast.

##### Cross-contamination by Peroxisomal Components

During this work, 15 proteins annotated as peroxisomal components were identified. Their functions range from lipid metabolism to oxidative stress and photorespiration (see supplemental Table S5, lanes 812–826). Experimental evidence from MASCP gator or SUBA show that most of these proteins are known peroxisome markers. As expected for abundant peroxisome components, some of these proteins were repeatedly detected in purified envelope, stroma, or thylakoid fractions (see supplemental Table S5, from column “ENV %” to column “Simm *et al.*, 2014”). From previous data analyzing the subcellular localizations of the members of the monodehydroascorbate reductase family (86), MDAR6 (At1g63940) was classified as a protein from the chloroplast stroma (EF = 0.4) and MDAR1 (At3g52880) was in peroxisomes (EF = 0.02). MDAR5 (mitochondria) and MDAR2 and 3 (cytosol) members were not detected during this work. Surprisingly, the MDAR4 member (At3g27820), previously detected in outer envelope membrane fractions purified from Pea chloroplasts (14), was also enriched (EF = 2.8) in our purified envelope fractions (see supplemental Table S5, lane 826). This MDAR4 isoform was previously shown to be localized in peroxisomes (transient expression of GFP fusions in tobacco or Arabidopsis cells), and to be the only membrane-associated member of the MDAR family thanks to a 60-residue extension located at its C terminus (86). Peroxisomes are highly motile organelles that can be detected close to chloroplasts, which suggests that they are physically interacting. Recently, Gao and colleagues (87) showed that chloroplasts and peroxisomes are physically tethered through peroxules, a poorly described structure in plant cells. Their observations suggest that peroxules might have a role in maintaining peroxisome-organelle interactions that could be important for fatty acid mobilization and photorespiration. The recent observations of these structures highlight a fundamentally important role for organelle interactions for essential biochemistry and physiological processes. It might also provide some explanation (alternative to cross-contamination) for the detection of some of these peroxisomal components in the purified envelope fractions during the present work.

Within the last two decades, many investigations of the chloroplast proteome have focused on either whole-chloroplast fractions or on independent subplastidial fractions (11, 12). Though highly informative, most of these databases do not contain accurate information about protein localization within the different subplastidial compartments. Therefore, the accurate localization of many envelope proteins remains mostly hypothetical. During this work, the use of MS-based parameters and manual annotation results in a strong correlation (see supplemental Table S7) between subcellular localizations deduced from our analyses and SUBAcon (88) or MASCP Gator (16). However, the present data complement these databases with information about the subplastidial locations of shared proteins. Now that MS sensitivity is enough to perform in-depth analysis of minor cell compartment proteomes and compare them with the proteomes of crude cell extracts, our approach should allow investigators to revisit other cell compartments. However, the success of these studies will rely on a prerequisite: only a strong enrichment factor will allow distinguishing minor components from cross-contaminants, *i.e.* abundant markers from other cell compartments.

To conclude, although work on chloroplast biology is progressing rapidly, the envelope compartment remains a hidden part of the organelle. Homology searches of many envelope proteins fail to identify homologues in other prokaryotic or eukaryotic species, suggesting that there are fundamental specificities in the controlled exchanges of ions, metabolites, proteins, metabolic pathways, and other mechanisms involved in the regulation of the morphology and the dynamics of higher plant chloroplasts. This is reflected in the results of our manual annotation in which many identified envelope proteins remain unknown or uncharacterized or were previously assigned unknown or erroneous subcellular/subplastidial localizations. Altogether, proteins identified here (see supporting supplemental Table S8) constitute a powerful resource that will help colleagues to delineate the principles and mechanisms controlling fundamental aspects of plastid biogenesis and functions.

## DATA AVAILABILITY

The mass spectrometry data have been deposited to the Wolverhampton Consortium via the PRIDE partner repository with the dataset identifier PXD010545 and 10.6019/PXD010545 (https://www.ebi.ac.uk/pride/archive/projects/PXD010545).

## Supplementary Material

supplemental Table S5

Supplemental Fig. 1 to 5

Supplemental table S1

Supplemental table S2

Supplemental table S3

Supplemental table S4

Supplemental table S5

Supplemental table S6

Supplemental table S7

Legends to supplemental data

Supplemental table S8

## Footnotes

^1^ The abbreviations used are:

EF: enrichment factor
MS: mass spectrometry
SC: spectral count
SSC: specific spectral count
WSC: weighted spectral count
SWSC: sum of weighted spectral count
CCE: crude cell extract
Env: envelope
IEM: inner envelope membrane
OEM: outer envelope membrane
THY: thylakoids
STR: Stroma.
