# Supplementary material for: Unraveling Hidden Components of the Chloroplast Envelope Proteome: Opportunities and Limits of Better MS Sensitivity
Source: Mol Cell Proteomics. 2019 Apr 8;18(7):1285–306. doi: 10.1074/mcp.RA118.000988 (PMC6601204; doi:10.1074/mcp.RA118.000988)
Supplement: supplemental Table S5 [file RA118.000988_index.html]

Supplement to Unravelling hidden components of the chloroplast envelope proteome: opportunities and limits of better MS sensitivity | Molecular & Cellular Proteomics

## Supplemental Data

- Supplemental Fig. 1 to 5 - Supplemental Fig. 1 to 5 with legends
- Supplemental table S1 - MS/MS identifications from analysis of Arabidopsis crude cell extracts (CCE1 to 3) and purified chloroplast envelope fractions (Env1 to 3).
- Supplemental table S2 - Oligonucleotides used to generate constructs (GFP and CFP fusions) to validate subcellular and subplastidial localization of TSP9, SFR2, UP1, eIF5A and VTE1 proteins
- Supplemental table S3 - List of proteins (2480) identified in crude cell extracts (2222) and purified Arabidopsis chloroplast envelope (1269) triplicates.
- Supplemental table S4 - Predicted subcellular localization of proteins identified in purified envelope fractions and crude cell extracts according to the SUBA3 database
- Supplemental table S5 - List of proteins identified in purified Arabidopsis chloroplast envelope triplicates
- Supplemental table S6 - Overlap of the 1269 proteins identified in purified envelope fractions with the list of 700 proteins previously identified in the envelope fractions and present in the AT\_CHLORO database
- Supplemental table S7 - Comparison of manual annotation (this work) with SUBAcon
- Legends to supplemental data - Legends to supplemental figures S1 to S5 and tables S1 to S8
- Supplemental table S8 - Simplified version of the table S5
