# Supplementary material for: Unraveling Hidden Components of the Chloroplast Envelope Proteome: Opportunities and Limits of Better MS Sensitivity
Source: Mol Cell Proteomics. 2019 Apr 8;18(7):1285–306. doi: 10.1074/mcp.RA118.000988 (PMC6601204; doi:10.1074/mcp.RA118.000988)
Supplement: Supplemental Fig. 1 to 5 [file 139688_2_supp_309958_ppdxw4.pdf]

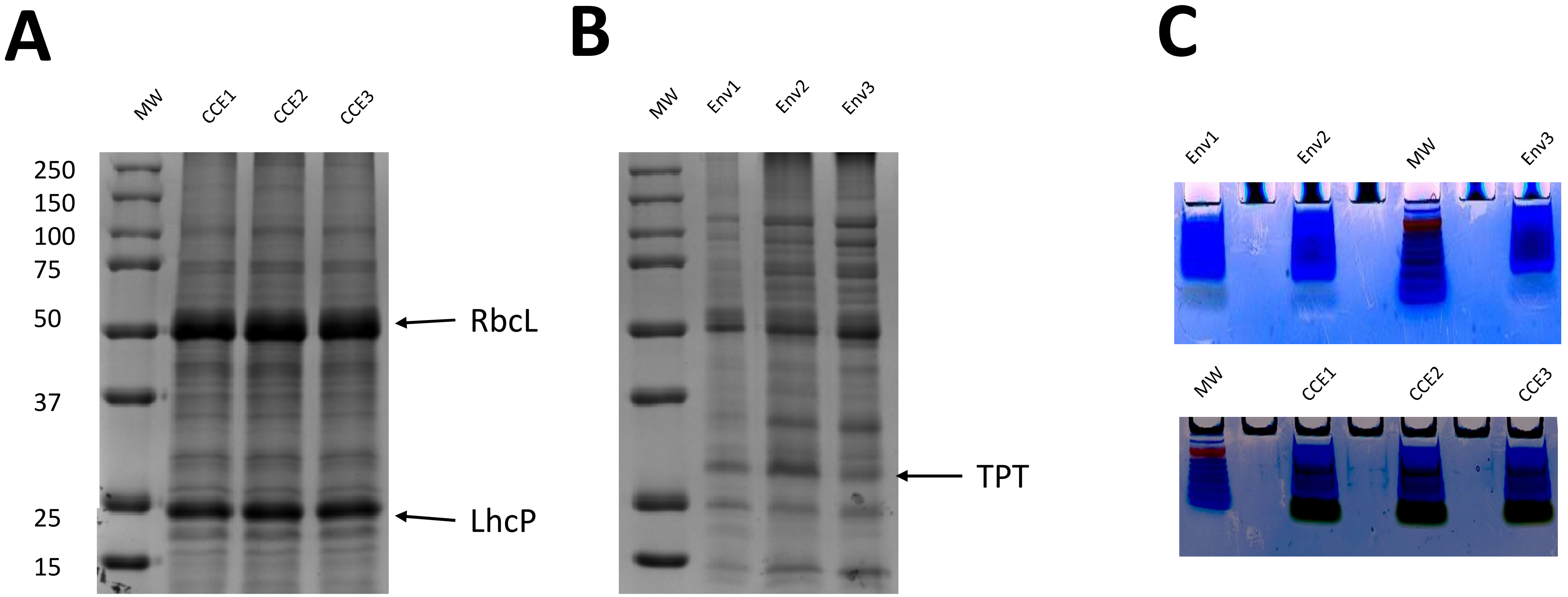

Supplemental Fig. S1: Similar protein profile over independent crude cell extracts and purified Arabidopsis chloroplast envelope fractions. The migration of the molecular weight standard (MW) is indicated on the left of each gel. Crude cell extract (CCE) (**A**) and purified chloroplast envelope (Env) (**B**) were prepared in triplicate as respectively indicated in lanes CCE1, CCE2, CCE3 and Env1, Env2, and Env3. Proteins (15 µg of each sample) were separated on a 12% SDS-PAGE further stained with Coomassie blue staining in order to assess reproducibility and homogeneity across triplicates. RBCL: Large subunit of Rubisco (marker from the stroma), TPT: Phosphate-triose-phosphate translocator (envelope marker) and LHCP: Light harvesting complex proteins (thylakoid marker) were detected in each sample. (**C**) Each protein sample (10 µg) was stacked by a 1 cm migration in the top of a NuPAGE 4–12% gel (Invitrogen) before Coomassie blue staining (R250, Bio-Rad). Gel bands of concentrated proteins were then manually excised from the gel and cut in pieces before protein digestion and further MS analysis.

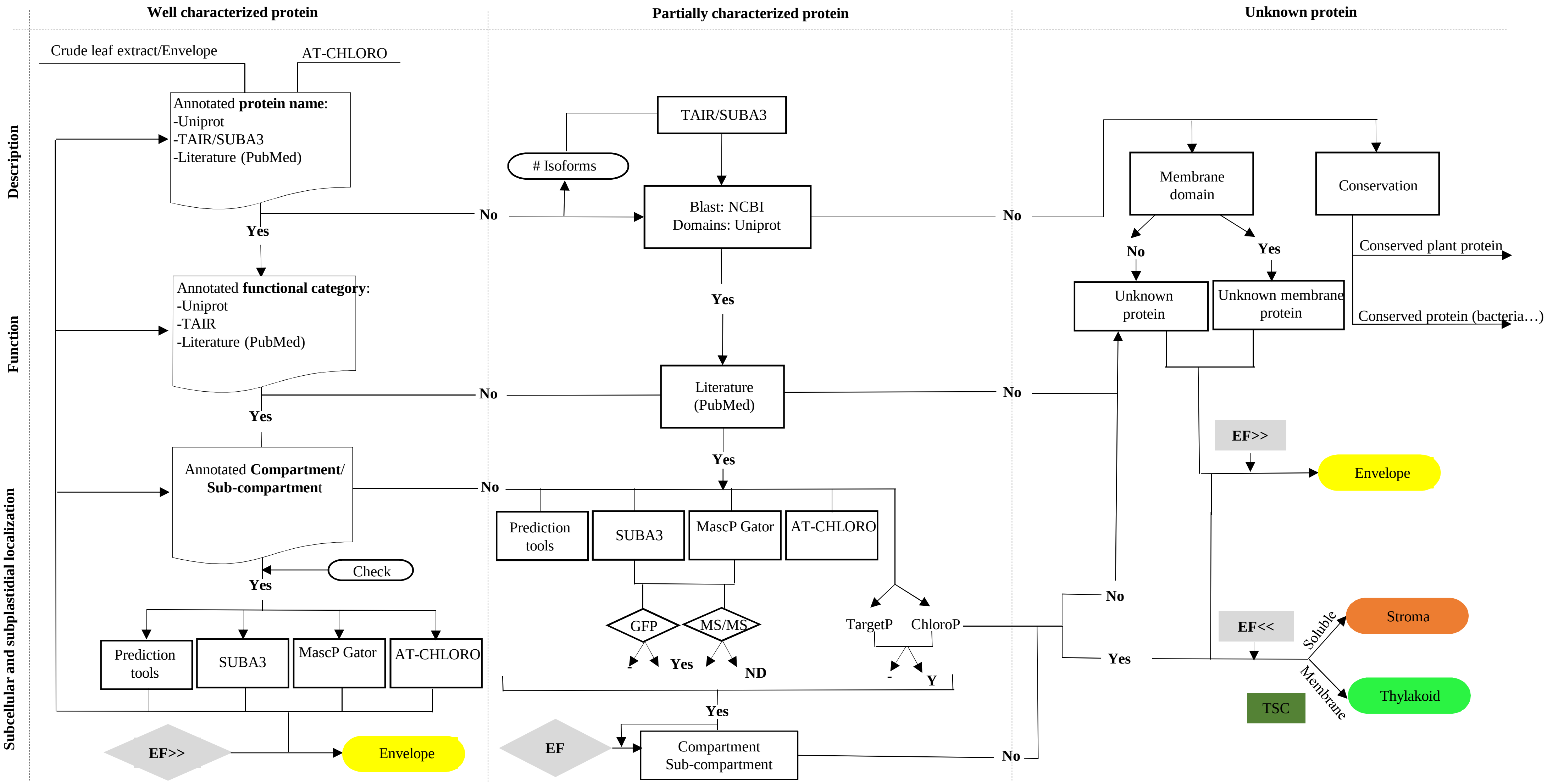

Supplemental Fig. S2: Overview of the manual annotation strategy. Subcellular and subplastidial localization, description, and functional annotation were manually performed using several databases and prediction tools. EF, the ratio of the sum of weighted spectral count (WSC) over each triplicate (EF estimator) was used to estimate the enrichment of each protein in the envelope fraction (E) compared to the total extract (CCE). This ratio was therefore used to differentiate genuine chloroplast envelope proteins from contaminants.

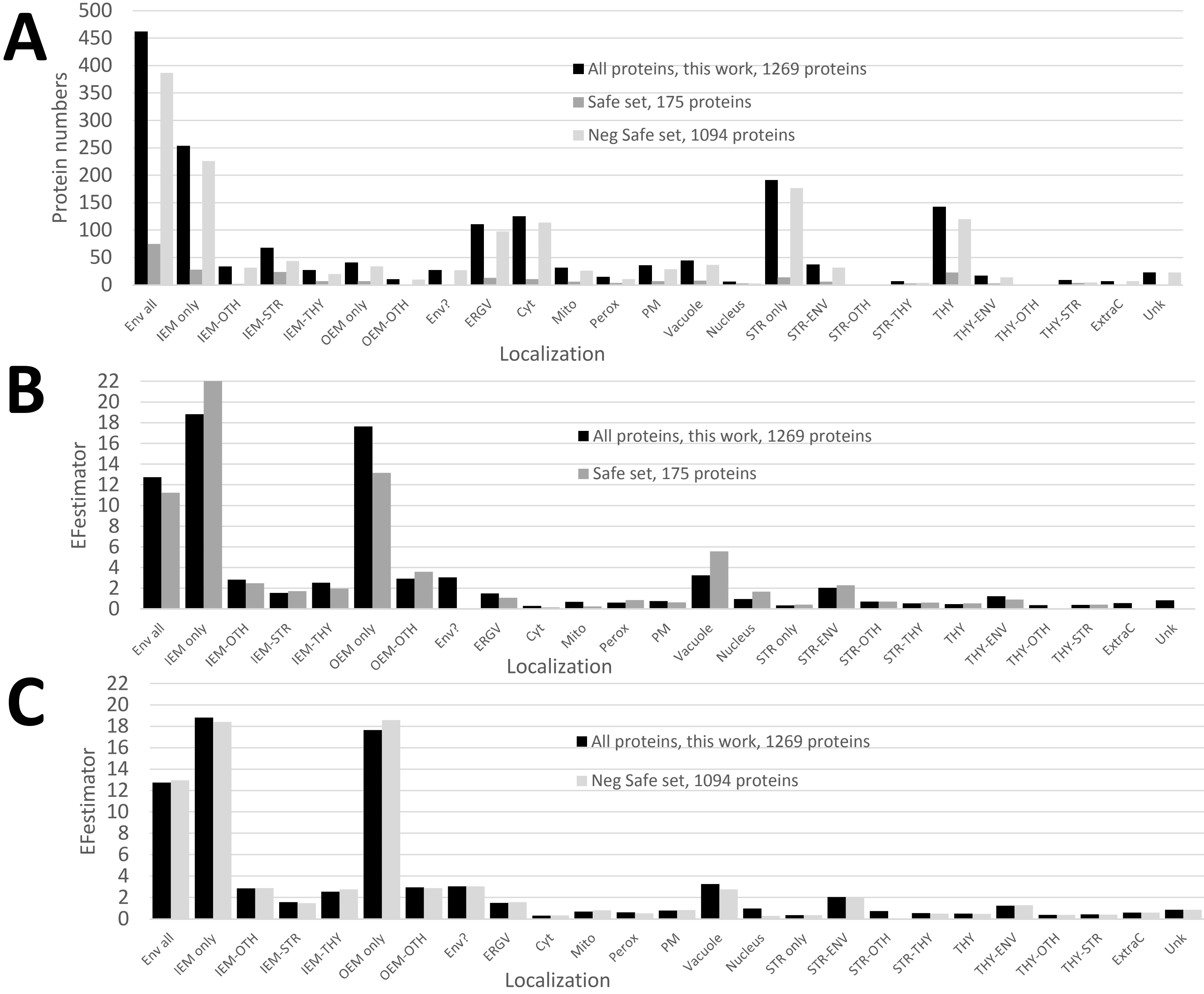

Supplemental Fig. S3: Validation of the EFestimator's ability to associate the 1269 proteins detected in purified envelope fractions with specific subcellular and subplastidial localizations. **A**. Numbers of proteins attributed to the various subplastidial and subcellular localizations. **B**. Average enrichment (EFestimator) of proteins attributed to the various subplastidial and subcellular localizations, forming a "safe set" of 175 proteins. **Black columns** indicate **all 1269 proteins** identified in purified envelope fractions (see supplemental table S5, "This work"). **Dark gray columns** indicate the **"Safe set",** *i.e.* the 175 proteins whose localizations were predicted from the consensus of all prediction tools in SUBA3 (SUBA.con) and confirmed using expression of GFP fusions in planta. Note that plastid-encoded or mitochondria-encoded proteins were also considered as "Safe" in their respective cell compartment. Also note that the 14 remaining proteins whose subplastidial location could not be deduced from AT_CHLORO were also removed from this "Safe set". **A and C. Light gray columns** indicate the **"Negative of the Safe set",** *i.e.* the 1094 proteins (1269 – 175) whose localizations were not strongly supported by previous data. Note that in both the **"Safe set"** and the **"Negative of the Safe set",** the enrichment of predicted envelope proteins is far above that of proteins associated with other plastid or cell compartments. Note the relatively low EF values of proteins that are shared between envelope and other plastid compartments, and the surprisingly high EF values of vacuolar proteins. IEM; Inner envelope membrane, OEM; outer envelope membrane, Env?; Envelope candidates; ERGV: endoplasmic reticulum/Golgi, Cyt; cytosol, Mito; mitochondria, Perox; peroxisome, PM; plasma membrane, STR; stroma, THY; thylakoid, ExtraC; extracellular, OTH; other, Unk; unknown and unpredictable localization.

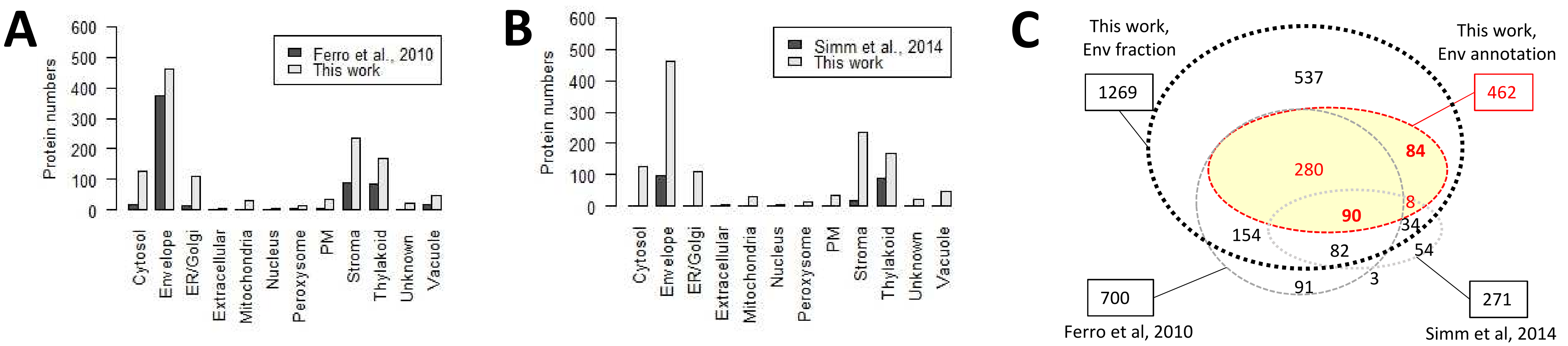

Supplemental Fig. S4: **Evaluation of coverage of the chloroplast envelope proteome when comparing present data with earlier analyses targeted to the same membrane system**. This work versus (**A**) Ferro et *al*. [12] or (**B**) Simm *et al*. [14]. Note that improved MS sensitivity allows detection of additional (probably minor) envelope proteins when compared to previous analyses. On the other hand, this is also the case for non-plastid compartments, cytosol and ER/Golgi proteins being specifically detected during this work. PM; plasma membrane. **C. Evaluation of coverage of the chloroplast envelope proteome when combining present data with earlier analyses targeted to the same membrane system.** Venn diagram indicating the weight of protein identified during this work when compared with previous data obtained by Ferro *et al*. (12) (see sup data 10 in (12)) or Simm *et al*. (14) (see sup data S8 in (14). Indicated values are numbers of proteins identified during the three studies. For the present analysis, we considered both the group of 1269 proteins identified in the purified envelope fractions and the group of 462 proteins classified as envelope components (thus excluding proteins classified as envelope "contaminants" and suspected to derive from non-plastid cell compartments (see supplemental table S5, "This work"). When combining all three studies, 84 envelope proteins were only identified during this work, 370 envelope proteins are shared between the present work and data obtained by Ferro *et al*. (12), but only 90 proteins were detected in all three studies. Note that most of the unique proteins identified by Ferro *et al*. (12) (*i.e.* 91 proteins) or Simm *et al*. (14) (*i.e.* 54 proteins) were detected in our crude cell extracts, and thus, assigned other subcellular or subplastidial localizations.

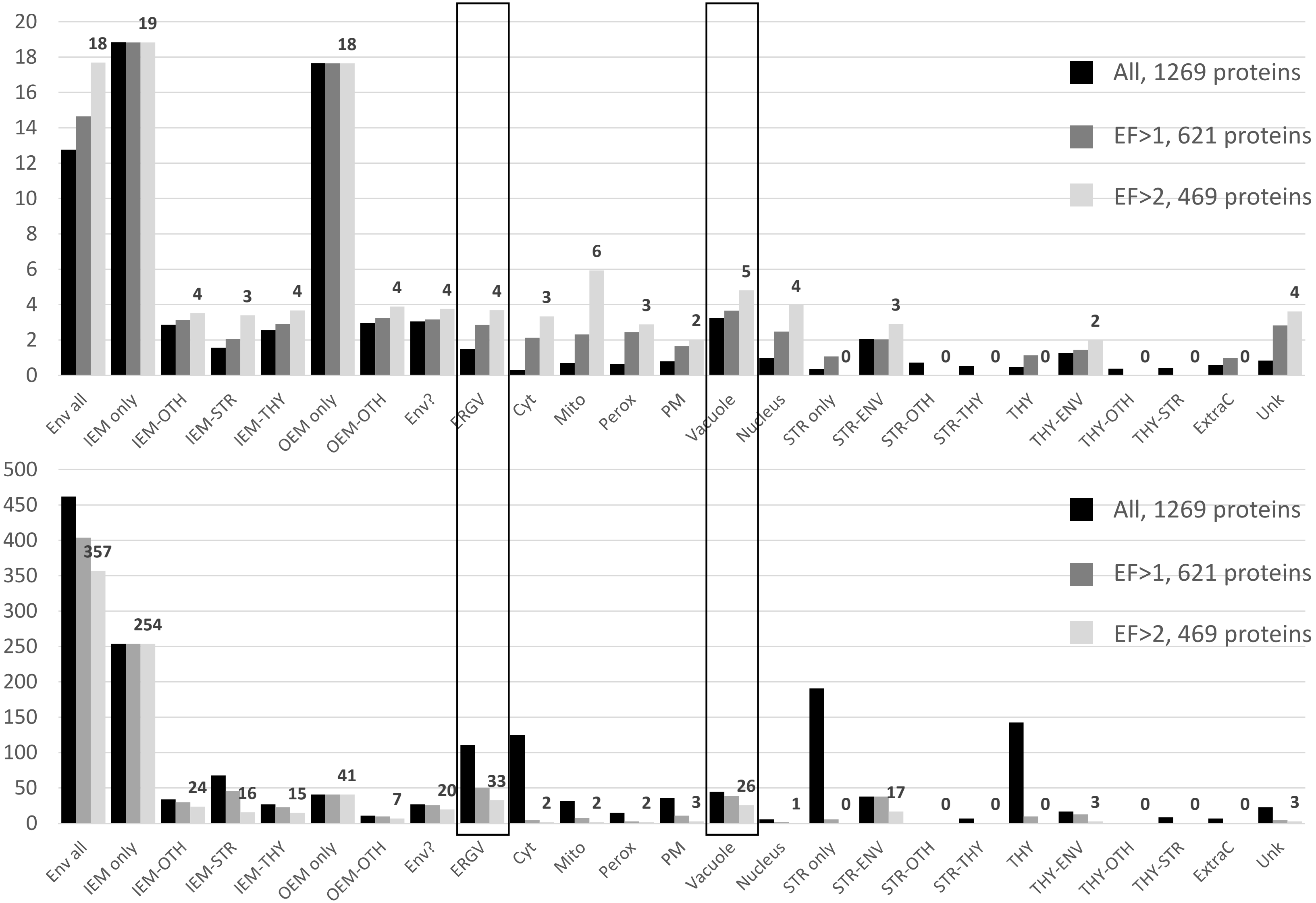

Supplemental Fig. S5: Impact of rising EF thresholds on the average EF values of proteins from other plastid and cell compartments. Note the high average EF values of the 33 remaining ER/Golgi (ERGV) and the 26 remaining vacuolar (Vacuole) proteins (black squares) when the EF threshold is increased to 2.
