# Supplementary material for: Unraveling Hidden Components of the Chloroplast Envelope Proteome: Opportunities and Limits of Better MS Sensitivity
Source: Mol Cell Proteomics. 2019 Apr 8;18(7):1285–306. doi: 10.1074/mcp.RA118.000988 (PMC6601204; doi:10.1074/mcp.RA118.000988)
Supplement: Supplemental table S2 [file 139688_2_supp_309960_ppdxwt.pptx]

## Slide 1
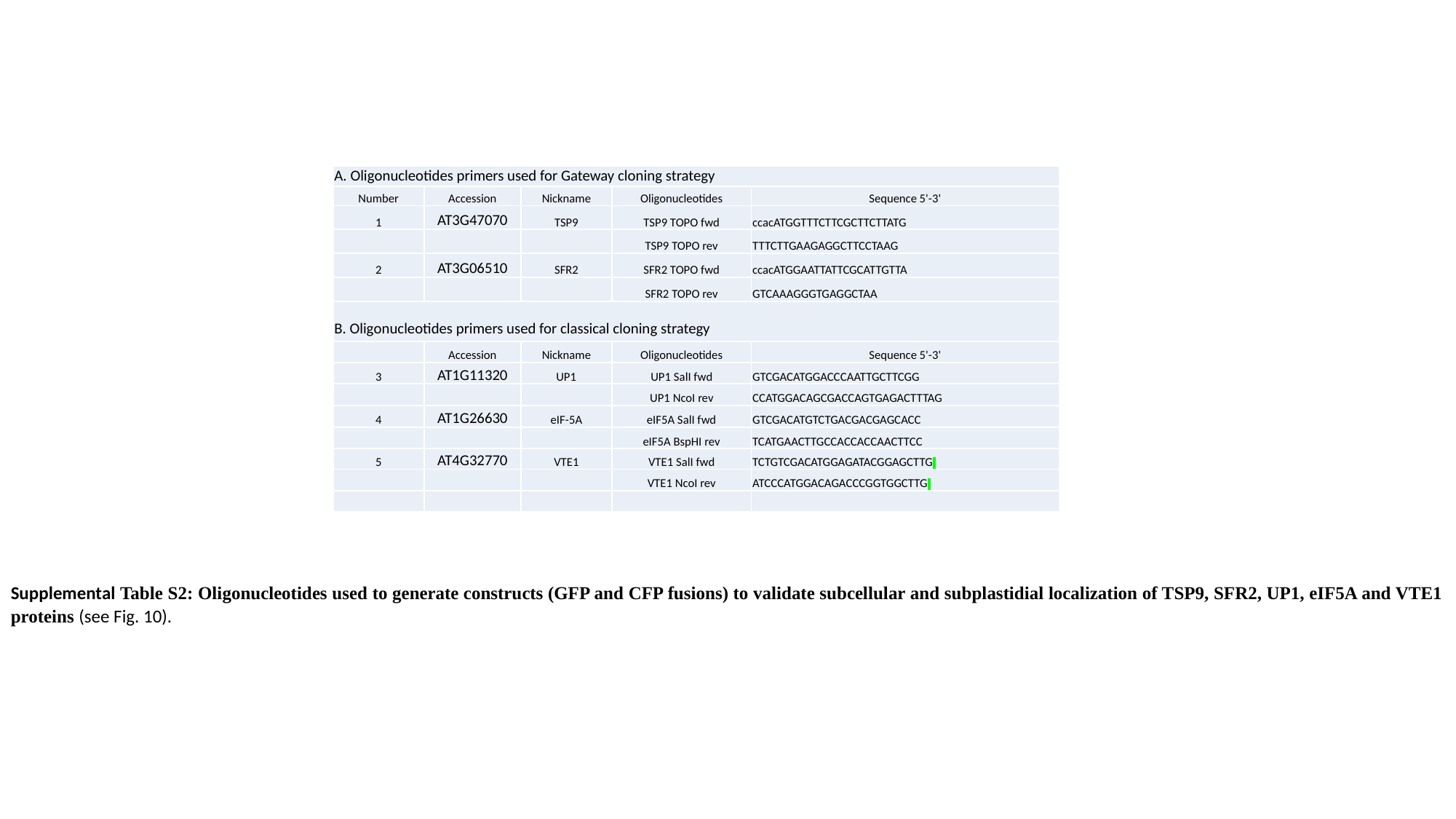

| A. Oligonucleotides primers used for Gateway cloning strategy | | | | |
| --- | --- | --- | --- | --- |
| Number | Accession | Nickname | Oligonucleotides | Sequence 5'-3' |
| 1 | AT3G47070 | TSP9 | TSP9 TOPO fwd | ccacATGGTTTCTTCGCTTCTTATG |
| | | | TSP9 TOPO rev | TTTCTTGAAGAGGCTTCCTAAG |
| 2 | AT3G06510 | SFR2 | SFR2 TOPO fwd | ccacATGGAATTATTCGCATTGTTA |
| | | | SFR2 TOPO rev | GTCAAAGGGTGAGGCTAA |
| B. Oligonucleotides primers used for classical cloning strategy | | | | |
| | Accession | Nickname | Oligonucleotides | Sequence 5'-3' |
| 3 | AT1G11320 | UP1 | UP1 SalI fwd | GTCGACATGGACCCAATTGCTTCGG |
| | | | UP1 NcoI rev | CCATGGACAGCGACCAGTGAGACTTTAG |
| 4 | AT1G26630 | eIF-5A | eIF5A SalI fwd | GTCGACATGTCTGACGACGAGCACC |
| | | | eIF5A BspHI rev | TCATGAACTTGCCACCACCAACTTCC |
| 5 | AT4G32770 | VTE1 | VTE1 SalI fwd | tctgtcgacatggagatacggagcttg |
| | | | VTE1 NcoI rev | atcccatggacagacccggtggcttg |
| | | | | |
Supplemental Table S2: Oligonucleotides used to generate constructs (GFP and CFP fusions) to validate subcellular and subplastidial localization of TSP9, SFR2, UP1, eIF5A and VTE1 proteins (see Fig. 10).
