# Supplementary material for: Unraveling Hidden Components of the Chloroplast Envelope Proteome: Opportunities and Limits of Better MS Sensitivity
Source: Mol Cell Proteomics. 2019 Apr 8;18(7):1285–306. doi: 10.1074/mcp.RA118.000988 (PMC6601204; doi:10.1074/mcp.RA118.000988)
Supplement: Supplemental table S4 [file 139688_2_supp_309962_ppdxwr.pptx]

## Slide 1
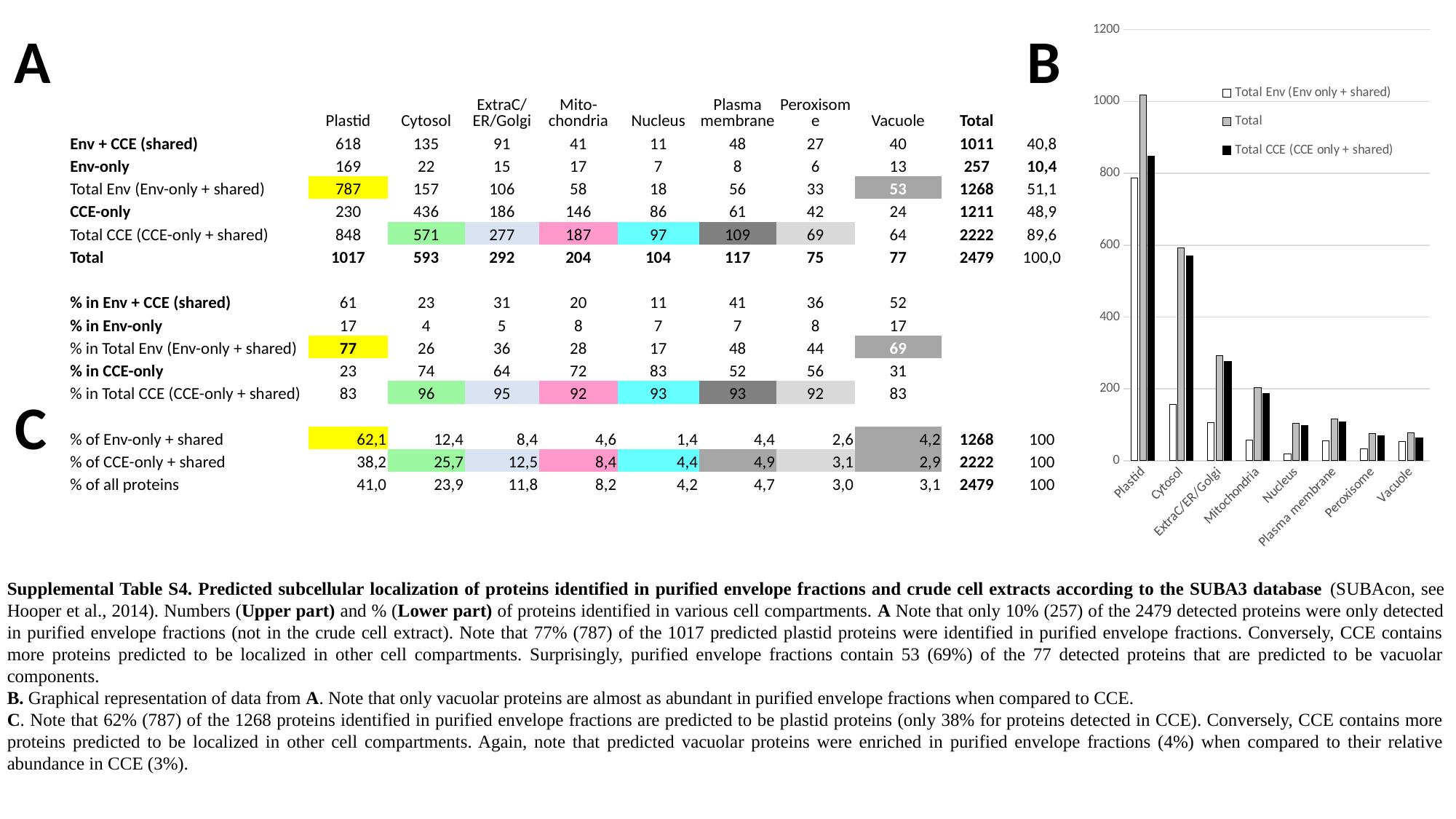

A
B
### Chart
| Category | Total Env (Env only + shared) | Total | Total CCE (CCE only + shared) |
|---|---|---|---|
| Plastid | 787.0 | 1017.0 | 848.0 |
| Cytosol | 157.0 | 593.0 | 571.0 |
| ExtraC/ER/Golgi | 106.0 | 292.0 | 277.0 |
| Mitochondria | 58.0 | 204.0 | 187.0 |
| Nucleus | 18.0 | 104.0 | 97.0 |
| Plasma membrane | 56.0 | 117.0 | 109.0 |
| Peroxisome | 33.0 | 75.0 | 69.0 |
| Vacuole | 53.0 | 77.0 | 64.0 || | Plastid | Cytosol | ExtraC/ER/Golgi | Mito-chondria | Nucleus | Plasma membrane | Peroxisome | Vacuole | Total | |
| --- | --- | --- | --- | --- | --- | --- | --- | --- | --- | --- |
| Env + CCE (shared) | 618 | 135 | 91 | 41 | 11 | 48 | 27 | 40 | 1011 | 40,8 |
| Env-only | 169 | 22 | 15 | 17 | 7 | 8 | 6 | 13 | 257 | 10,4 |
| Total Env (Env-only + shared) | 787 | 157 | 106 | 58 | 18 | 56 | 33 | 53 | 1268 | 51,1 |
| CCE-only | 230 | 436 | 186 | 146 | 86 | 61 | 42 | 24 | 1211 | 48,9 |
| Total CCE (CCE-only + shared) | 848 | 571 | 277 | 187 | 97 | 109 | 69 | 64 | 2222 | 89,6 |
| Total | 1017 | 593 | 292 | 204 | 104 | 117 | 75 | 77 | 2479 | 100,0 |
| | | | | | | | | | | |
| % in Env + CCE (shared) | 61 | 23 | 31 | 20 | 11 | 41 | 36 | 52 | | |
| % in Env-only | 17 | 4 | 5 | 8 | 7 | 7 | 8 | 17 | | |
| % in Total Env (Env-only + shared) | 77 | 26 | 36 | 28 | 17 | 48 | 44 | 69 | | |
| % in CCE-only | 23 | 74 | 64 | 72 | 83 | 52 | 56 | 31 | | |
| % in Total CCE (CCE-only + shared) | 83 | 96 | 95 | 92 | 93 | 93 | 92 | 83 | | |
| | | | | | | | | | | |
| % of Env-only + shared | 62,1 | 12,4 | 8,4 | 4,6 | 1,4 | 4,4 | 2,6 | 4,2 | 1268 | 100 |
| % of CCE-only + shared | 38,2 | 25,7 | 12,5 | 8,4 | 4,4 | 4,9 | 3,1 | 2,9 | 2222 | 100 |
| % of all proteins | 41,0 | 23,9 | 11,8 | 8,2 | 4,2 | 4,7 | 3,0 | 3,1 | 2479 | 100 |
C
Supplemental Table S4. Predicted subcellular localization of proteins identified in purified envelope fractions and crude cell extracts according to the SUBA3 database (SUBAcon, see Hooper et al., 2014). Numbers (Upper part) and % (Lower part) of proteins identified in various cell compartments. A Note that only 10% (257) of the 2479 detected proteins were only detected in purified envelope fractions (not in the crude cell extract). Note that 77% (787) of the 1017 predicted plastid proteins were identified in purified envelope fractions. Conversely, CCE contains more proteins predicted to be localized in other cell compartments. Surprisingly, purified envelope fractions contain 53 (69%) of the 77 detected proteins that are predicted to be vacuolar components.
B. Graphical representation of data from A. Note that only vacuolar proteins are almost as abundant in purified envelope fractions when compared to CCE.
C. Note that 62% (787) of the 1268 proteins identified in purified envelope fractions are predicted to be plastid proteins (only 38% for proteins detected in CCE). Conversely, CCE contains more proteins predicted to be localized in other cell compartments. Again, note that predicted vacuolar proteins were enriched in purified envelope fractions (4%) when compared to their relative abundance in CCE (3%).
