# Supplementary material for: Unraveling Hidden Components of the Chloroplast Envelope Proteome: Opportunities and Limits of Better MS Sensitivity
Source: Mol Cell Proteomics. 2019 Apr 8;18(7):1285–306. doi: 10.1074/mcp.RA118.000988 (PMC6601204; doi:10.1074/mcp.RA118.000988)
Supplement: Supplemental table S7 [file 139688_2_supp_309965_ppdxwc.pptx]

## Slide 1
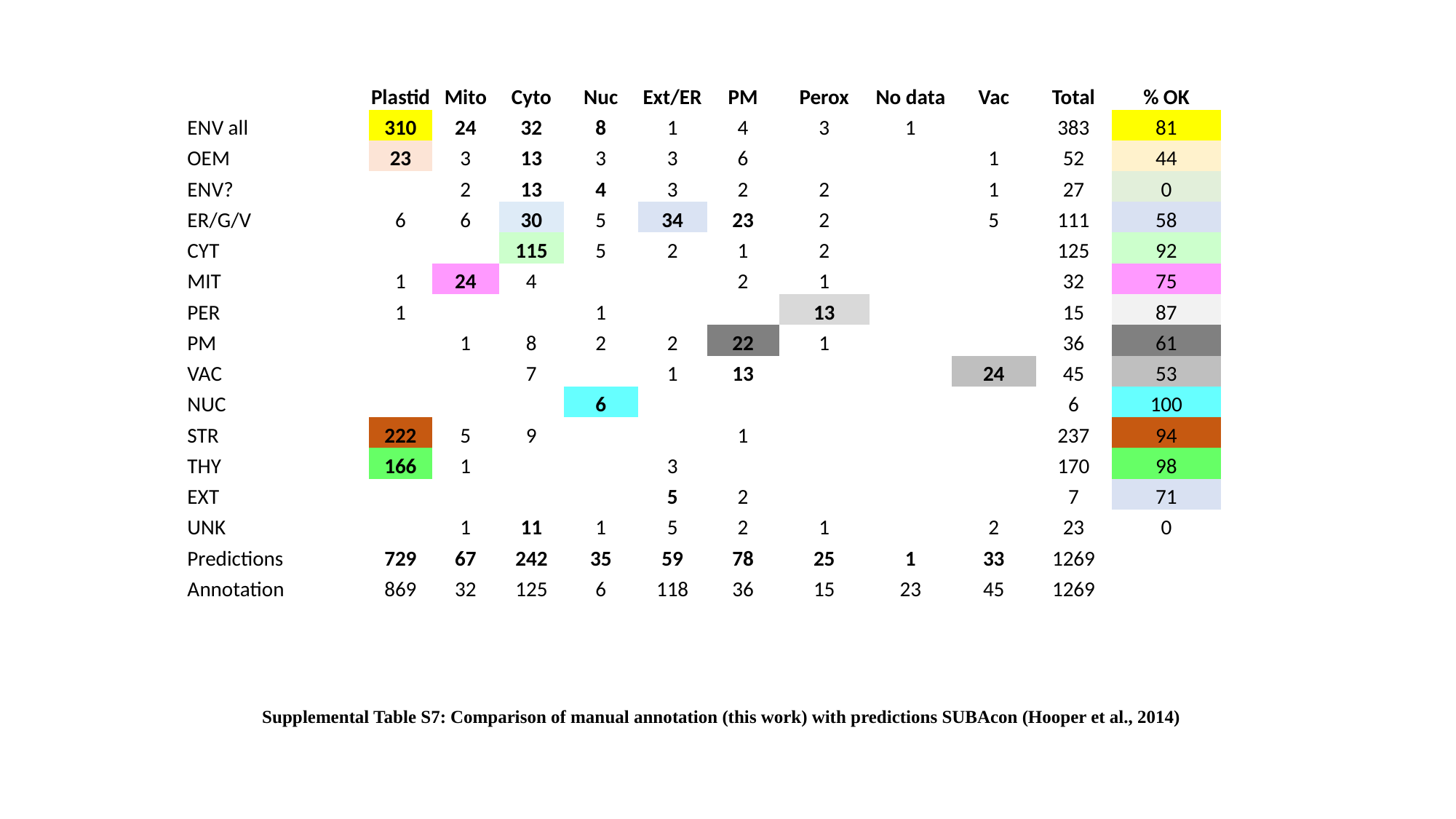

| | | | | | | | | | | | | | |
| --- | --- | --- | --- | --- | --- | --- | --- | --- | --- | --- | --- | --- | --- |
| | | | Plastid | Mito | Cyto | Nuc | Ext/ER | PM | Perox | No data | Vac | Total | % OK |
| ENV all | | | 310 | 24 | 32 | 8 | 1 | 4 | 3 | 1 | | 383 | 81 |
| OEM | | | 23 | 3 | 13 | 3 | 3 | 6 | | | 1 | 52 | 44 |
| ENV? | | | | 2 | 13 | 4 | 3 | 2 | 2 | | 1 | 27 | 0 |
| ER/G/V | | | 6 | 6 | 30 | 5 | 34 | 23 | 2 | | 5 | 111 | 58 |
| CYT | | | | | 115 | 5 | 2 | 1 | 2 | | | 125 | 92 |
| MIT | | | 1 | 24 | 4 | | | 2 | 1 | | | 32 | 75 |
| PER | | | 1 | | | 1 | | | 13 | | | 15 | 87 |
| PM | | | | 1 | 8 | 2 | 2 | 22 | 1 | | | 36 | 61 |
| VAC | | | | | 7 | | 1 | 13 | | | 24 | 45 | 53 |
| NUC | | | | | | 6 | | | | | | 6 | 100 |
| STR | | | 222 | 5 | 9 | | | 1 | | | | 237 | 94 |
| THY | | | 166 | 1 | | | 3 | | | | | 170 | 98 |
| EXT | | | | | | | 5 | 2 | | | | 7 | 71 |
| UNK | | | | 1 | 11 | 1 | 5 | 2 | 1 | | 2 | 23 | 0 |
| Predictions | | | 729 | 67 | 242 | 35 | 59 | 78 | 25 | 1 | 33 | 1269 | |
| Annotation | | | 869 | 32 | 125 | 6 | 118 | 36 | 15 | 23 | 45 | 1269 | |
| | | | | | | | | | | | | | |
Supplemental Table S7: Comparison of manual annotation (this work) with predictions SUBAcon (Hooper et al., 2014)
