# Supplementary material for: Unraveling Hidden Components of the Chloroplast Envelope Proteome: Opportunities and Limits of Better MS Sensitivity
Source: Mol Cell Proteomics. 2019 Apr 8;18(7):1285–306. doi: 10.1074/mcp.RA118.000988 (PMC6601204; doi:10.1074/mcp.RA118.000988)
Supplement: Legends to supplemental data [file 139688_2_supp_309966_ppb5qb.pdf]

LEGENDS TO SUPPLEMENTAL DATA

Legends to Supplemental Figures

Supplemental Fig. S1: Similar protein profile over independent crude cell extracts and purified Arabidopsis chloroplast envelope fractions. The migration of the molecular weight standard (MW) is indicated on the left of each gel. Crude cell extract (CCE) **(A)** and purified chloroplast envelope (Env) **(B)** were prepared in triplicate as respectively indicated in lanes CCE1, CCE2, CCE3 and Env1, Env2, and Env3. Proteins (15 µg of each sample) were separated on a 12% SDS-PAGE further stained with Coomassie blue staining in order to assess reproducibility and homogeneity across triplicates. RBCL: Large subunit of Rubisco (marker from the stroma), TPT: Phosphate-triose-phosphate translocator (envelope marker) and LHCP: Light harvesting complex proteins (thylakoid marker) were detected in each sample. **(C)** Each protein sample (10 µg) was stacked by a 1 cm migration in the top of a NuPAGE 4–12% gel (Invitrogen) before Coomassie blue staining (R250, Bio-Rad). Gel bands of concentrated proteins were then manually excised from the gel and cut in pieces before protein digestion and further MS analysis.

Supplemental Fig. S2: Overview of manual annotation strategy. Subcellular and subplastidial localization, description, and functional annotation were manually performed using several databases and prediction tools. EF, the ratio of the sum of weighted spectral count (WSC) over each triplicate (EF estimator) was used to estimate the enrichment of each protein in the envelope fraction (E) compared to the total extract (CCE). This ratio was therefore used to differentiate genuine chloroplast envelope proteins from contaminants.

Supplemental Fig. S3: Validation of the EFestimator's ability to associate the 1269 proteins detected in purified envelope fractions with specific subcellular and subplastidial localizations. A. Numbers of proteins attributed to the various subplastidial and subcellular localizations. **B.** Average enrichment (EFestimator) of proteins attributed to the various subplastidial and subcellular localizations, forming a "safe set" of 175 proteins. **Black columns** indicate all **1269 proteins** identified in purified envelope fractions (see supplemental table S5, "This work"). **Dark gray columns** indicate the "**Safe set**", *i.e.* the 175 proteins whose localizations were predicted from the consensus of all prediction tools in SUBA3 (SUBAcon) and confirmed using expression of GFP fusions *in planta*. Note that plastid-encoded or mitochondria-encoded proteins were also considered as "Safe" in their respective cell compartment. Also note that the 14 remaining proteins whose subplastidial location could not be deduced from AT_CHLORO were also removed from this "Safe set". **A and C. Light gray columns** indicate the "**Negative of the Safe set**", *i.e.* the 1094 proteins (1269 – 175) whose localizations were not strongly supported by previous data. Note that in both the "**Safe set**" and the "**Negative of the Safe set**", the enrichment of predicted envelope proteins is far above that of proteins associated with other plastid or cell compartments. Note the relatively low EF values of proteins that are shared between envelope and other plastid compartments, and the surprisingly high EF values of vacuolar proteins. IEM; Inner envelope membrane, OEM; outer envelope membrane, Env?; Envelope candidates; ERGV: endoplasmic reticulum/Golgi, Cyt; cytosol, Mito; mitochondria, Perox; peroxisome, PM; plasma membrane, STR; stroma, THY; thylakoid, ExtraC; extracellular, OTH; other, Unk; unknown and unpredictable localization.

Supplemental Fig. S4 (former Fig. 4): **Evaluation of coverage of the chloroplast envelope proteome when comparing present data with earlier analyses targeted to the same membrane**

system. This work versus (A) Ferro et *al*. (12) or (B) Simm *et al*. (14). Note that improved MS sensitivity allows detection of additional (probably minor) envelope proteins when compared to previous analyses. On the other hand, this is also the case for non-plastid compartments, cytosol and ER/Golgi proteins being specifically detected during this work. PM; plasma membrane. **C. Evaluation of coverage of the chloroplast envelope proteome when combining present data with earlier analyses targeted to the same membrane system.** Venn diagram indicating the weight of protein identified during this work when compared with previous data obtained by Ferro *et al*. (12) (see sup data 10 in (12)) or Simm *et al*. (14) (see sup data S8 in (14)). Indicated values are numbers of proteins identified during the three studies. For the present analysis, we considered both the group of 1269 proteins identified in the purified envelope fractions and the group of 462 proteins classified as envelope components (thus excluding proteins classified as envelope "contaminants" and suspected to derive from non-plastid cell compartments (see supplemental table S5, "This work"). When combining all three studies, 84 envelope proteins were only identified during this work, 370 envelope proteins are shared between the present work and data obtained by Ferro *et al*. (12), but only 90 proteins were detected in all three studies. Note that most of the unique proteins identified by Ferro *et al*. (12) (*i.e.* 91 proteins) or Simm *et al*. (14) (*i.e.* 54 proteins) were detected in our crude cell extracts, and thus, assigned other subcellular or subplastidial localizations.

Supplemental Fig. S5 (former Supplemental Fig. S4): Impact of rising EF thresholds on the average EF values of proteins from other plastid and cell compartments. Note the high average EF values of the 33 remaining ER/Golgi and the 26 remaining vacuolar proteins (black squares) when the EF threshold is increased to 2.

Legends to Supplemental Tables

Supplemental table S1: MS/MS identifications from analysis of Arabidopsis crude cell extracts (CCE1 to 3) and purified chloroplast envelope fractions (Env1 to 3). The lists of proteins and peptides result from an automated validation pipeline as described in materials and methods. The corresponding mass spectrometry data are available at the PRIDE repository (23) with the dataset identifier PXD010545.

Protein sets: list of all protein sets (2964 including 16 contaminants) identified in the whole experiment. Proteins retained must have at least one SSC in one replicate. "sameset" and "subset" accessions indicate proteins that share the same set of peptides, or a subset of the peptides, respectively. Note that the “Protein sets” are tagged “DISCARDED” or “SELECTED” in this worksheet, according to additional filters applied manually: *i*) contaminants (keratin…) were discarded. *ii*) protein groups detected with only one specific peptide that were partially filtered by excluding proteins having a total WSC sum = 1 over the whole experiment. **Protein matches**: list of all (representative, sameset and subset) proteins belonging to protein sets (5562 including 136 contaminants). **Best PSMs**: list of best (highest score) Peptide Spectrum Matches of peptides identified. Unique peptides (15460) are defined by a unique combination of primary sequences + modifications and positions.

Supplemental table S2: Oligonucleotides used to generate constructs (GFP and CFP fusions) to validate subcellular and subplastidial localization of TSP9, SFR2, UP1, eIF5A and VTE1 proteins (see Fig. 8).

A. Oligonucleotides primers used for Gateway cloning strategy

Number	Accession	Nickname	Oligonucleotides	Sequence 5'-3'
1	AT3G47070	TSP9	TSP9 TOPO fwd	*ccac*ATGGTTTCTTCGCTTCTTATG
			TSP9 TOPO rev	TTTCTTGAAGAGGCTTCCTAAG
2	AT3G06510	SFR2	SFR2 TOPO fwd	*ccac*ATGGAATTATTCGCATTGTTA
			SFR2 TOPO rev	GTCAAAGGGTGAGGCTAA

B. Oligonucleotides primers used for classical cloning strategy

	Accession	Nickname	Oligonucleotides	Sequence 5'-3'
3	AT1G11320	UP1	UP1 SalI fwd	GTCGACATGGACCCAATTGCTTCGG
			UP1 NcoI rev	CCATGGACAGCGACCAGTGAGACTTTAG
4	AT1G26630	eIF-5A	eIF5A SalI fwd	GTCGACATGTCTGACGACGAGCACC
			eIF5A BspHI rev	TCATGAACTTGCCACCACCAACTTCC
5	AT4G32770	VTE1	VTE1 SalI fwd	TCTGTCGACATGGAGATACGGAGCTTG
			VTE1 NcoI rev	ATCCCATGGACAGACCCGGTGGCTTG

Supplemental table S3: List of proteins (2480) identified in crude cell extracts (2222) and purified *Arabidopsis* chloroplast envelope (1269) triplicates. Crude cell extract (CCE1 to 3) and purified chloroplast envelope fractions (Env1 to 3) were prepared in triplicates as indicated in materials and methods. Composition of these triplicates were analyzed by MS. Accession: AGI numbers. Description: annotations extracted from databases. Sequence specific: number of sequences not shared by another protein set. Pep: number of identified peptides. SC: spectral counts. SSC: specific spectral counts. WSC: weighted spectral counts. WSC_CCE: ~~specific~~ weighted spectral counts in crude cell extracts. WSC_Env: weighted spectral counts in envelope fractions. Norm_WSC_CCE, normalized WSC over the sum of replicate in crude cell extracts. Norm_WSC_Env, normalized WSC over the sum of replicate in envelope fractions. Location SUBAcon: known or predicted subcellular localization extracted from SUBA3 (see (86)) to design Fig. 2. Description SUBA3: protein description extracted from the SUBA3 database.

Supplemental table S4. Predicted subcellular localization of proteins identified in purified envelope fractions and crude cell extracts according to the SUBA3 database (SUBAcon, see (86)). Numbers (**Upper part)** and % (**Lower part)** of proteins identified in various cell compartments. **A** Note that only 10% (257) of the 2479* detected proteins were only detected in purified envelope fractions. Note that 77% (787) of the 1017 predicted plastid proteins were identified in purified envelope fractions. Conversely, CCE contains more proteins predicted to be localized in other cell compartments. Surprisingly, purified envelope fractions contain 53 (69%) of the 77 detected proteins that are predicted to be vacuolar components.

B. Graphical representation of data from **A**. Note that only vacuolar proteins are nearly as abundant in purified envelope fractions when compared to CCE. **C**. Note that 62% (787) of the 1269 proteins identified in purified envelope fractions are predicted to be plastid proteins (only 38% for proteins detected in CCE). Conversely, CCE contains more proteins predicted to be localized in other cell compartments. Again, note that predicted vacuolar proteins were enriched in purified envelope fractions (4%) when compared to their relative abundance in CCE (3%). * Note that one protein (AT5G12170.2) has no SUBAcon.

Supplemental table S5: List of proteins identified in purified *Arabidopsis* chloroplast envelope triplicates. All proteins: manual annotation of the 1364 non-redundant proteins identified in purified envelope fractions during this work and in Ferro *et al*., 2010 (12). **This work:** list of the 1269 proteins identified in purified envelope fractions during this work. **Only EF>1**: list of the 641 proteins identified in purified envelope fractions with a WSC_Env/WSC_CCE ratio > 1. **Only EF>2**: list of the 469 proteins identified in purified envelope fractions, with a WSC_Env/WSC_CCE ratio > 2.

Protein group (AGI numbers), Protein number according to Sup Data AT_CHLORO (12), accession number (AGI numbers), accession in UNIPROT, classification in MapManBin (-sept 2017), Curated function, curated description. Simplified location AT_CHLORO: subplastidial localization in AT_CHLORO (12) (nd: not detected in Ferro *et al*., 2010 (12)). Simplified location This work: revised subplastidial localization according to new manual annotation. WSC_CCE: weighted spectral counts in crude cell extracts. WSC_Env: weighted spectral counts in envelope fractions. WSC_Env/WSC_CCE: ratio of weighted spectral counts in envelope fractions to weighted spectral counts in crude cell extracts. Env %, STR % and THY % refer to detection of the protein in the three main chloroplast compartments in AT_CHLORO (12). TSC refers to the detection of the protein in thylakoid sub-compartments (89). TargetP: prediction of subcellular localization (29). cTP: prediction of chloroplast localization using ChloroP (28). cTP (loc): predicted maturation site according to ChloroP (28). Env proteome: detection of the protein by Simm *et al*., 2014 (14). Experimental evidence MASCP Gator / SUBA3: detection of the protein using MS-based approaches in specific cell compartments. Predicted MASCP Gator & SUBA: numbers of independent tools predicting specific subcellular localizations. Location GFP (MASCP GATOR & SUBA3): experimental evidences for the specific subcellular localization of a protein. Location SUBAcon: consensus subcellular localization of the protein extracted from SUBA3 (86). Sequence Specific in ENV: number of specific sequences detected in purified envelope fractions (or only in CCE for “All proteins” since some proteins detected in Ferro *et al*., 2010 (12) were only detected in CCE during this work). Crude cell extract (CCE_T1 to T3) and purified chloroplast envelope fractions (Env_E1 to E3) are triplicates, as indicated in materials and methods. Norm_WSC, normalized Weighted Spectral Count. SWSC, sum of the Norm_WSC in each sample. Imp_SWSC, imputed SWSC *i.e.* when this sum equals zero in the CCE fraction, an extra count of 1 was added to both SWSC_Env and SWSC_CCE.

Supplemental Table S6: **Overlap of the 1269 proteins identified in purified envelope fractions with the list of 700 proteins previously identified in the envelope fractions and present in the AT_CHLORO database** (12) (see supplemental table S10 of (12)).

Supplemental Table S7: **Comparison of manual annotation (this work) with SUBAcon** ((88))

Supplemental Table S8: **Simplified version of Supplemental table S5 as a quick reference of the "reannotated envelope proteome" for non-expert plant biologists.**
